# Adaptation strategies of endolithic chlorophototrophs to survive the hyperarid and extreme solar radiation environment of the Atacama Desert

**DOI:** 10.3389/fmicb.2015.00934

**Published:** 2015-09-10

**Authors:** Jacek Wierzchos, Jocelyne DiRuggiero, Petr Vítek, Octavio Artieda, Virginia Souza-Egipsy, Pavel Škaloud, Michel Tisza, Alfonso F. Davila, Carlos Vílchez, Inés Garbayo, Carmen Ascaso

**Affiliations:** ^1^Museo Nacional de Ciencias Naturales, CSICMadrid, Spain; ^2^Biology Department, The Johns Hopkins UniversityBaltimore, MD, USA; ^3^Laboratory of Ecological Plant Physiology, Global Change Research Centre AS CRBrno, Czech Republic; ^4^Institute of Geochemistry, Mineralogy and Mineral Resources, Charles UniversityPrague, Czech Republic; ^5^Departamento Biología Vegetal, Ecología y Ciencias de la Tierra, Universidad de ExtremaduraPlasencia, Spain; ^6^Instituto de Ciencias Agrarias, CSICMadrid, Spain; ^7^Department of Botany, Charles UniversityPrague, Czech Republic; ^8^Carl Sagan Center, SETI InstituteMountain View, CA, USA; ^9^Facultad de Ciencias Experimentales, Universidad de HuelvaHuelva, Spain

**Keywords:** Atacama Desert, carotenoids, endolithic chlorophototrophs, extreme environment, gypsum, scytonemin

## Abstract

The Atacama Desert, northern Chile, is one of the driest deserts on Earth and, as such, a natural laboratory to explore the limits of life and the strategies evolved by microorganisms to adapt to extreme environments. Here we report the exceptional adaptation strategies of chlorophototrophic and eukaryotic algae, and chlorophototrophic and prokaryotic cyanobacteria to the hyperarid and extremely high solar radiation conditions occurring in this desert. Our approach combined several microscopy techniques, spectroscopic analytical methods, and molecular analyses. We found that the major adaptation strategy was to avoid the extreme environmental conditions by colonizing cryptoendolithic, as well as, hypoendolithic habitats within gypsum deposits. The cryptoendolithic colonization occurred a few millimeters beneath the gypsum surface and showed a succession of organized horizons of algae and cyanobacteria, which has never been reported for endolithic microbial communities. The presence of cyanobacteria beneath the algal layer, in close contact with sepiolite inclusions, and their hypoendolithic colonization suggest that occasional liquid water might persist within these sub-microhabitats. We also identified the presence of abundant carotenoids in the upper cryptoendolithic algal habitat and scytonemin in the cyanobacteria hypoendolithic habitat. This study illustrates that successful lithobiontic microbial colonization at the limit for microbial life is the result of a combination of adaptive strategies to avoid excess solar irradiance and extreme evapotranspiration rates, taking advantage of the complex structural and mineralogical characteristics of gypsum deposits—conceptually called “rock's habitable architecture.” Additionally, self-protection by synthesis and accumulation of secondary metabolites likely produces a shielding effect that prevents photoinhibition and lethal photooxidative damage to the chlorophototrophs, representing another level of adaptation.

## Introduction

In the inhospitable environment of extremely arid deserts, microbial life has found refuge in very specific microhabitats (Pointing and Belnap, 2012; Wierzchos et al., 2012a; de los Ríos et al., 2014a). One of these microhabitats is the endolithic habitat, which consists of a network of pores and fissures connected to the surface within translucent rock (Nienow, 2009). Endolithic colonization can be viewed as a stress avoidance strategy, where the overlying mineral substrate provides efficient protection from incident lethal UV radiation and excessive photosynthetically active radiation, thermal buffering, protection from freeze–thaw events, physical stability and enhanced moisture availability (Walker and Pace, 2007; Pointing and Belnap, 2012; Wierzchos et al., 2012a; de los Ríos et al., 2014a). Remarkable examples of lithic habitats are found in the hyperarid zone of the Atacama Desert, in northern Chile. These habitats are as diverse as the insides of halite (Wierzchos et al., 2006; de los Ríos et al., 2010; Stivaletta et al., 2012; Robinson et al., 2015), gypsum (Dong et al., 2007; Wierzchos et al., 2011; DiRuggiero et al., 2013; Vítek et al., 2013; Ziolkowski et al., 2013a), carbonate rocks (DiRuggiero et al., 2013) and volcanic rocks (Wierzchos et al., 2013). The Atacama Desert is known as one of the driest places on our Planet with scarce precipitations as low as 3 mm y^−1^ in its hyperarid core (Houston and Hartley, 2003; McKay et al., 2003; Hartley et al., 2005; Houston, 2006a,b). However, hyperaridity is not solely due to a lack of rainfall (P); it is also related to potential evapotranspiration (PET) and may be defined as a ratio of P/PET of less than 0.05 according to United Nations Environment Program. By this definition, virtually the whole area of the Atacama Desert, between 15 and 30°S and at elevations up to 3500 m a.s.l., may be considered as hyperarid (Houston and Hartley, 2003).

In addition to its extreme dryness, the Atacama Desert holds records for other extreme environmental characteristics. The highest surface ultraviolet (UV) radiations and total solar irradiances ever measured on Earth have been reported from its high altitude “Altiplano” area (Piacentini et al., 2003; Cordero et al., 2014) and pre-Andean Domeyko Cordillera (Rondanelli et al., 2015). The prevalent cloudless conditions and relatively low total ozone column in the Atacama Desert result in extremely high solar radiation, increasing considerably the probability of lethal photoinhibition and photooxidative damage to phototrophs (Solovchenko and Merzlyak, 2008). Adaptation strategies for protection against high solar irradiance were observed in several microorganisms from the Atacama Desert. For example, the thallus of the lichen *Acarospora schleicheri*, which is found in the Altiplano area of Chile, accumulates rhizocarpic acid upon exposure to high levels of UV-B radiation (Rubio et al., 2002). Biomineralization of amorphous silica (sinter) around cyanobacteria filaments has been reported as a significant irradiation shield from the El Tatio geothermal field at 4300 m a.s.l. in the Andes Mountains (Phoenix et al., 2006). A different strategy to avoid stressful light conditions was found within the endolithic habitat of halite (NaCl) rocks. The *Halothece* cyanobacteria (Wierzchos et al., 2006; de los Ríos et al., 2010; Robinson et al., 2015), a unique cyanobacteria colonizing halites synthesizes large amounts of scytonemin, a well-known UV protective molecule (Vítek et al., 2010, 2012, 2014).

When both extreme desiccation and high ambient UV fluxes are combined, such as in areas of the hyperarid core of the Atacama Desert, the surface of rocks can be rendered sterile (Cockell et al., 2008). Under these extreme conditions, a millimeter-thick layer of gypsum has been found sufficient to protect microorganisms from UV-induced killing (Cockell et al., 2008). Microporous and translucent gypsum formations have been reported as lithic substrate harboring endolithic microbial communities in different climates such as moderate (Boison et al., 2004), polar and subarctic (Hughes and Lawley, 2003; Parnell et al., 2004; Edwards et al., 2005; Cockell et al., 2010; Ziolkowski et al., 2013b; Rhind et al., 2014), dry and arid (Stivaletta and Barbieri, 2009; Stivaletta et al., 2010), and dry and hyperarid (Dong et al., 2007; Wierzchos et al., 2011; DiRuggiero et al., 2013; Vítek et al., 2013; Ziolkowski et al., 2013a). In many of the above studies, translucent gypsum was reported to allow photosynthesis while providing protection from excess solar irradiance and retaining sufficient moisture for microorganisms survival. These factors may account for the high biodiversity found in gypsum-hosted communities (see Supplementary Table S1 for a summary of gypsum endolithic communities). However, there is a lack of knowledge regarding the microorganisms vertical organization or the distribution of photosynthetic pigments and secondary metabolites within endolithic community. No studies have addressed the effect of light gradients, or other characteristics of the lithic substrate, on the spatial localization of the different microbial taxa inhabiting this extreme environment.

The aim of the present work was to elucidate exceptional adaptation strategies of chlorophototrophic (i.e., chlorophyll-based phototrophs) microorganisms colonizing the insides of gypsum deposits in the hyperarid zone of the Atacama Desert. These endolithic microbial colonies are taking advantage of the physical and structural properties of translucent gypsum deposits containing sepiolite inclusions. Using a combination of microscopic and spectroscopic analytical methods, molecular and phylogenetic analyses, and micromorphological studies, we provide a detailed characterization of the chlorophototrophic microorganisms colonizing the gypsum endolithic habitat. Together with long-term microclimatic data and the composition and structure of the mineral substrate, we constructed a conceptual model of the vertical succession of microorganisms, together with the distribution of their photosynthetic pigments and secondary metabolites, within gypsum deposits under extremes of aridity and solar radiation.

## Materials and methods

The following is the summary of methods used in this study. More detailed information is provided in Supplementary Materials.

### Site characterization and sampling

**Figure 1:**
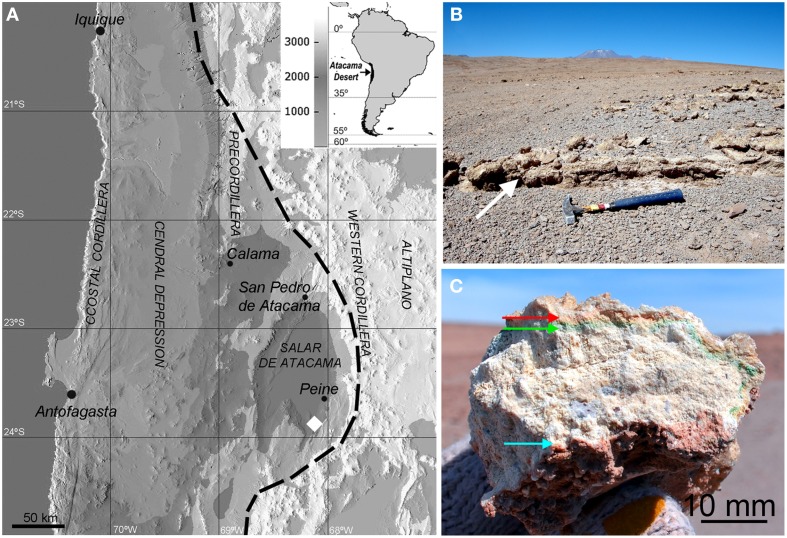
**(A)** Shaded relief digital elevation map (DEM) showing the sampling area south of the Salar de Atacama basin (white diamond) and the geomorphic units of the central Atacama Desert; dashed line is the limit of the hyperarid area (Houston and Hartley, 2003). **(B)** Sampling area showing gypsum deposits (white arrow); the Socompa volcano is seen on the horizon. **(C)** Cross section of gypsum bearing cryptoendolithic (red and green arrows) and hypoendolithic (blue arrow) microbial colonization.

The sampling zone (23°53′S, 068°08′W and 2720 m a.s.l.) was located in the south of the Salar de Atacama basin (Figure 1A) in the north-south trending depression of the Cordon de Lila range, in northern Chile. This depression is mostly covered by volcanic ash material but large gypsum outcrops (1 × 2 m) can be found in several locations (Figure 1B). In the field, gypsum deposits were assessed for endolithic colonization by visual inspection of microbial pigments present in fractured samples. These pigments indicate the presence of cryptoendolithic (occupying pore spaces beneath rock surface, Nienow, 2009) and hypoendolithic (colonizing the undermost layer of the rock, sensu Wierzchos et al., 2011) microbial colonization forming horizons beneath the surface and close to the bottom of the gypsum deposits (Figure 1C). Colonized gypsum samples (10 × 10 cm) were collected in 2013 in the Cordon de Lila area, sealed in sterile Whirlpacks®, and stored at room temperature in a dark and dry environment until analysis.

### Environmental data acquisition

Microclimate data were collected 6 km from the sampling site for 39 months (37 for T and RH), from January 20th, 2010 to April 20th, 2013, using an Onset HOBO® Weather Station Data Logger (H21-001) connected to a SolarStream® solar-powered transmitter for data transmission by Iridium Satellite Constellation. Sensors recorded measurements every 30 min intervals. Air relative humidity (RH) and temperature (T) were recorded 25 cm above the rock surface (probe was shaded from the sun) using RH/T sensors (HOBO® S-THB-M002; precision, ±2.5% RH and ±0.2°C T). Solar flux was measured using a photosynthetically active radiation (PAR) sensor for wavelengths of 400–700 nm. PAR data were also indicative of cloud cover at the sampling site. Rainfall was monitored using a Rain-o-Matic 100 (PRONAMIC ApS, Herning, Denmark) tipping bucket gauge (resolution of 1 mm). The presence of liquid water on the gypsum surface was determined by electrical conductivity (EC) using sensor as described by Wierzchos et al. (2013) (see also Supplementary Section S1).

### Mineralogy X-ray diffraction analyses

The mineralogical composition of the gypsum duricrust was identified by X-ray powder diffraction (XRD) using a Philips X'Pert diffractometer (Philips, Amsterdam, The Netherlands) with graphite-monochromated CuKα radiation (see detailed procedures in Supplementary Section S2).

### Petrographic microscopy

Petrography studies of gypsum duricrust thin sections (30 μm-thick) were conducted using a Nikon Eclipse LV100Pol (Nikon, Tokyo, Japan) polarized light microscope equipped with a Nikon DS-Fi1 digital camera.

### Mercury intrusion porosimetry

Pore space and pore size distribution of the gypsum samples were characterized by mercury intrusion porosimetry (MIP). Three types of subsamples were analyzed: samples from the surface dense crust (above the colonization zone), samples from the interior of the gypsum deposits (within the colonization zone), and samples from the bottom dense crust (covering the hypoendolithic colonization zone). A PoreMaster 60/Quantachrome (Quantachrome Instruments, Boynton Beach, USA) instrument was used to determine porosity (connected porosity) of the rock samples in the pore diameter range 0.0036–190.58 μm. A mercury surface tension value of 0.48 N/m and rock-mercury contact angle of 140 dag were used in the Washburn or Laplace equations, respectively.

### Measurements of light conditions within gypsum deposits

The photosynthetic photon fluence rate (PPFR) was measured beneath the gypsum surface at different depths (up to 1.5 mm) with a small (3.5 mm in diameter) spherical sensor. These measurements were performed on wetted gypsum after simulation of a 10 mm rainfall to measure light conditions, inside the rock, when the presence of water would allow photosynthetic activity. Measurements for UVA and UVB radiation beneath the gypsum surface at different depths (up to 1.5 mm) were performed using cosine corrected sensors. Detailed procedures are provided in Supplementary Section S3.

### Fluorescence microscopy (FM)

Small fragments of gypsum showing distinct signs of endolithic colonization with a green/orange layer beneath the rock surface and a green layer just above the bottom of the gypsum sample were scraped and suspended in double-distilled water. Suspensions were vortexed (2 min) and allowed to settle for 1 min before removing the supernatant, which was centrifuged for 10 min at 5000 g. The pellet was then stained with SYBR Green I (SBI) (Molecular Probes), a fluorocromes used for the specific staining of nucleic acids. Bright field images, SBI fluorescence and photosynthetic pigment autofluorescence were visualized in a Zeiss AxioImager D1 fluorescence microscope (Carl Zeiss, Jena, Germany). Detailed procedures are provided in Supplementary Section S4.

### Scanning electron microscopy in backscattered electron mode (SEM-BSE)

Colonized gypsum samples were processed for SEM-BSE and energy dispersive X-ray spectroscopy (EDS) microanalysis as described in Wierzchos et al. (2011). As the intensity of the BSE signal depends on the mean atomic number of the sample, the SEM-BSE technique identifies heavy metal-stained ultrastructural elements of microbial cells. SEM-BSE was then used in combination with EDS to characterize the minerals associated with specific cell aggregates. Gypsum samples were observed using a scanning electron microscope (DSM960 Zeiss, Oberkochen, Germany) equipped with a solid-state, four diodes BSE detector plus an auxiliary X-ray EDS microanalytical system (Link ISIS Oxford, UK).

### Transmission electron microscopy (TEM)

Fragments of colonized gypsum material were processed for transmission electron microscopy. Ultrathin sections were visualized using EM910 (Leo, Oberkochen, Germany), CM200 Philips (Philips, Amsterdam, The Nederlands) and analyzed using energy dispersive X-ray spectroscopy (EDS) microanalysis. Detailed procedures are provided in Supplementary Section S5.

### Low temperature scanning electron microscopy

We characterize the features of gypsum deposits and internal micromorphology of microbial cells in their natural hydrated state using low temperature scanning electron microscope (LT-SEM) according to procedures described by Wierzchos et al. (2012b). The LT-SEM (DSM960 Zeiss, Oberkochen, Germany) was equipped with a cryotransfer system (CT1500, Oxford, UK) and with imaging systems using secondary electrons (SE) and backscattered electrons (BSE) detectors, and with an X-ray energy dispersive spectroscopy (EDS) system.

### Algal culturing and micromorphological characterization

Fragments of gypsum samples bearing the green/orange colored cryptoendolithic colonization were visualized by stereoscopic microscope with aim to find algal colonies. Small rock fragments inhabited by algae were mechanically scrapped off into Petri dishes poured with agarized BBM (Deason and Bold, 1960) and BG-11 mineral media (Stanier et al., 1971). Algae were inoculated at 20°C under a 14 h/10 h light/dark regime using photon irradiance of approximately 30–50 μmol photons m^−2^ s^−1^ provided by 18 W cool fluorescent tubes (Philips TLD 18W/33). After 5–10 weeks, the agar plates were checked for the presence of algal microcolonies. The algae were investigated using an Olympus BX51 (Olympus, Tokyo, Japan) light microscope with differential interference contrast (DIC). Microphotographs were taken with an Olympus Z5060 digital camera.

### Microbial community analyses

Environmental DNA was extracted from the top colonization zone of two individual gypsum rocks using the PowerSoil DNA isolation kit (MoBio laboratories Inc., Solana Beach, CA) as described in Robinson et al. (2015). DNA was amplified using the barcoded universal primers 27F and 506R for the V1–V3 hypervariable region of the 16S rRNA gene and amplicons from 3 reactions were pooled together for sequencing using the Illumina MiSeq platform by the Genomics Resource Center (GRC) at the Institute for Genome Sciences (IGS), University of Maryland School of Medicine (Robinson et al., 2015). We obtained a total of 3326 paired-end reads for two rock samples (CL1 and CL2) collected from the same location, with an average size of 436 bp. The QIIME package (v1.6.0) was used to process the Illumina paired-end reads as previously described (Robinson et al., 2015). Sequence reads were normalized to 1662 reads per sample and diversity metrics were calculated based on OTUs at the 0.03% cutoff in QIIME (Caporaso et al., 2010). Nonparametric Mann-Whitney tests were used for statistical analysis. We use the following 18S rRNA primer sets to obtain nuclear sequences from the algae: 82F-1498R (Bachy et al., 2011); ChloroF-ChloroR and BaciF-BaciR (Valiente Moro et al., 2009); Euk328F-Euk329R (Romari and Vaulot, 2004); EUK528f-CHLO02r (Zhu et al., 2005).

### Analyses of photosynthetic pigments by raman spectroscopy

Cryptoendolithic and hypoendolithic colonization zones, harboring phototrophic eukaryotes and prokaryotes, were examined using point Raman analysis on a Thermo Fisher DXR Raman microspectrometer (Thermo Scientific, Waltham, USA) with a 532 nm diode laser and a Renishaw InVia Reflex Raman microspectrometer using both 785 nm diode and 514.5 nm Ar laser. The later Raman system was used to construct Raman distribution maps of carotenoids within the algal zone using 514.5 nm laser line. The analyses were performed on freshly cut transects of the gypsum substrate. The Raman spectroscopy technique was chosen for its *in situ* approach with small amount of biological material. Detailed procedures are provided in Supplementary Section S6.

### Spectrophotometric analyses

The absorption spectra of acetone extracted photosynthetic and light-protecting pigments (chlorophyll a and b, and carotenoids) were obtained using a HP 8452A Diode Array dual-beam spectrophotometer (Hewlett-Packard, Tokyo, Japan). Absorbance values for characteristic wavelength (maximum peaks for particular pigments) were selected for semiquantitative determination of pigments contents [mg/g of the dry weight (DW) in powdered sample] using the trichromatic equations and extinction coefficients (Lichtenthaler, 1987). Detailed methodology sample processing and pigment extraction are provided in Supplementary Section S7.

## Results

### Microclimate data

**Figure 2:**
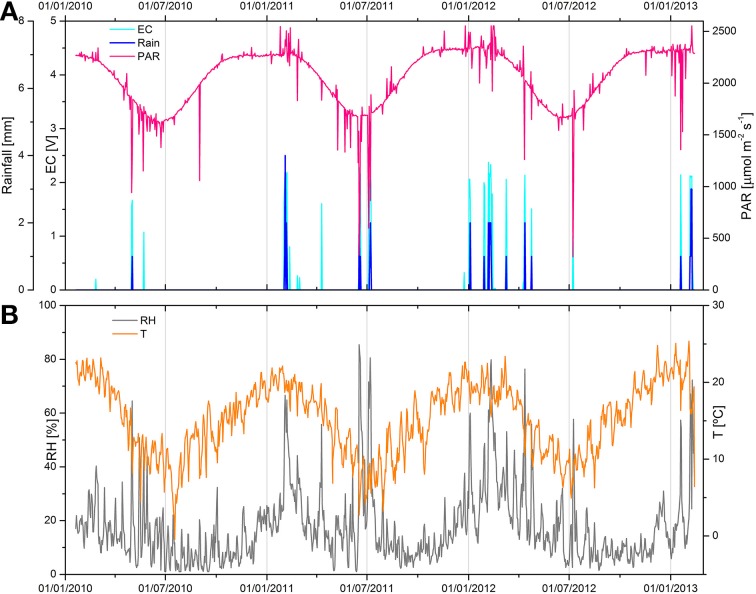
**Microclimate data collected from January 20th, 2010 to February 20th, 2013 nearby the Cordon de Lila sampling site. (A)** Maximum daily photosynthetically active radiation (PAR, purple line), rainfall precipitations (blue bars indicating mm of rainfall), and cyan bars indicating moisture episodes identified by increase in electrical conductivity (EC) values. **(B)** Medium daily temperature (T, orange line) and medium daily relative humidity (RH, gray line) oscillations.

Microclimatic parameters were recoded with a microweather station in close proximity to the sampling site over 39 months (37 for T and RH measurements), starting in January 2010. The maximum daily photosynthetically active radiation, rainfall precipitations, and electrical conductivity measured on the gypsum gypcrete surface, as well as mean the daily air relative humidity and mean daily air temperature oscillation measured at the sampling site are shown in Figure 2. Some of this data (first 22 months) was previously reported by Wierzchos et al. (2013). PAR values revealed a very intense solar irradiance over the 3-year period of recording (Figure 2A). The mean daily PAR value was 1175 μmol photons m^−2^ s^−1^ and during the austral summer (January, *n* = 4) the mean maximum daily value was as high as 2298 μmol photons m^−2^ s^−1^. For a number of days, usually in February, spikes of extremely high PAR values, up to 2554 μmol photons m^−2^ s^−1^, were recoded (Figure 2A). These spikes likely originated from light scattering by cumulus cloud formation (Piacentini et al., 2003), although cloudy days were very scarce.

The sum of recorded rainfall precipitation over the three-year period was 88 mm and represented 19 separate episodes (Figure 2A). The annual mean precipitation was 27.1 mm y^−1^ with 61% of rain precipitations occurring during the summer months (January-March). The maximum daily rain precipitation was observed on 17th June 2011 with a total of 14 mm of rain between 8:30 a.m. and 11:00 p.m. EC sensors detected liquid water on the gypsum surface mostly in the late evening and until about midnight (Figure 2A). Most of these events corresponded to rainfall episodes with the exception of 7 events where moisture was detected by EC readings with no rainfall gauge reading. These 7 events could be the result of intermittent rainfalls below the detection limit of rainfall gauge (< 1 mm). Moisture on the rock surface persisted several hours after the rainfall (see Supplementary Figure S1). The total time of moisture at the rock surface, as detected by the EC sensor, was 638 h for the 39 months periods, giving a value of 196 h y^−1^ (about 8 days y^−1^). These values represent surface water and it is likely that moisture was available inside the gypsum rock for longer periods. Time periods between moisture events were exceptionally long with a maximum of 254 days in 2010. Analyses of our microclimate data indicated that rainfall was the only source of liquid water potentially available for the gypsum microbial communities.

Oscillation of mean daily air temperatures was in accordance with seasonal changes of solar irradiance (Figure 2B). The mean T for the 37 month period was 15.3°C. The mean daily maximum T in summer (January-February 2013) was 43.6°C with a maximum of 49.3°C. The minimum recorded T was −7.4°C. Air RH values were remarkably low over the entire recording period. Mean annual RH for 3 years was as low as 16.5% with mean winter and spring seasonal RH values between 7 and 11%. RH above 80–90% was only observed during short and infrequent rainfall episodes.

### Mineralogical and physical characterization of gypsum-composed gypcrete

**Figure 3:**
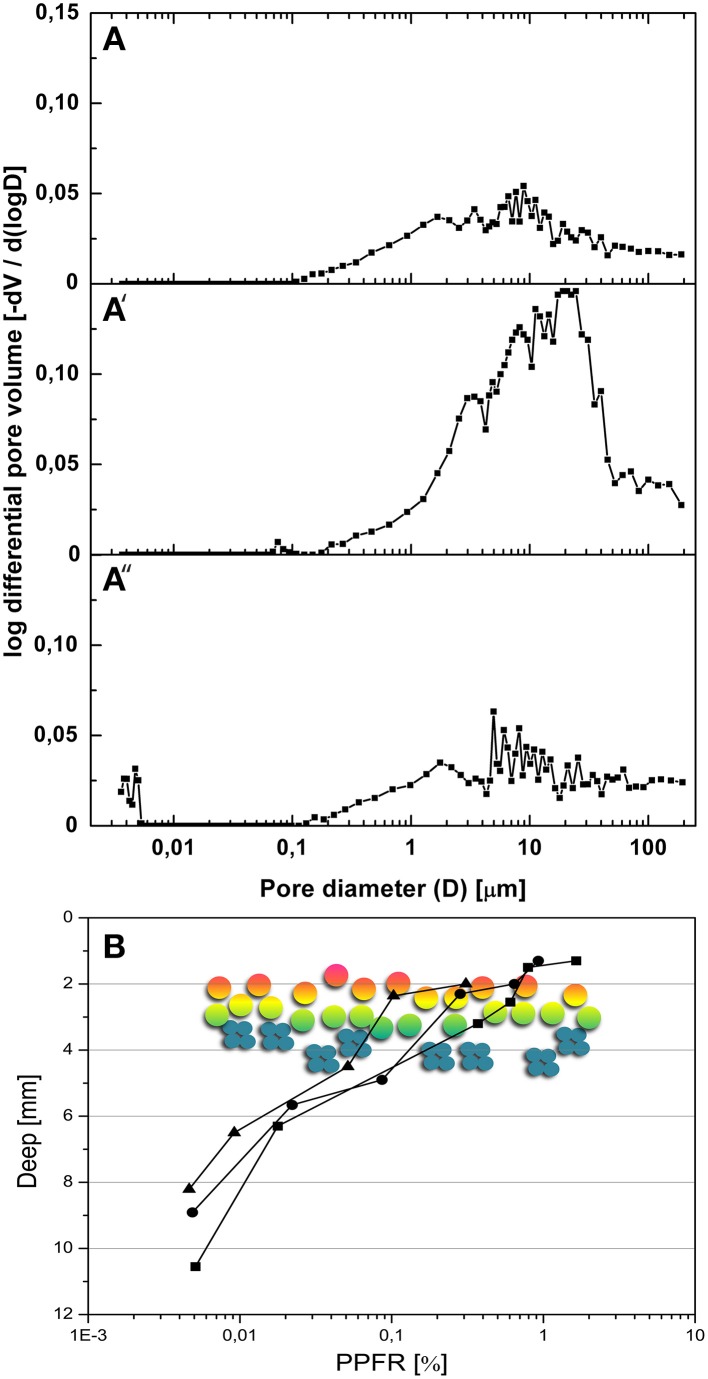
**Porosity and light transmission properties of gypsum deposits. (A–A″)** pore size distribution curves for surface layer **(A)**, interior **(A**′**)** and bottom layer **(A**″**)**; mean curves for four samples. **(B)** Intensity of photosynthetic photon fluence rate (PPFR) within gypsum wet deposits at different depths (*n* = 3); hypothetical position of cryptoendolithic colonization zone is shown by the schematic positioning of green-orange algae (circles) and cyanobacteria (small cyan discs).

Gypsum formations at the Cordon de Lila sampling site appear as hard layer deposits on the soil surface, interbedded between layers of ignimbrites and/or filling large cracks on these rocks. Volcanic ashes cemented by gypsum have been previously reported in this region of the Atacama Desert (Bao, 2005). This type of formation is called gypcrete according to the nomenclature by Horta (1980). The XRD analysis of Ca-sulfate deposits revealed gypsum (CaSO4·2H_2_O) as the major mineral (96–98%) with minor amounts of cristobalite (SiO_2_), calcite (CaCO_3_), and potassium feldspar (KAlSi_3_O_8_). A clay mineral with X-ray reflection at 12.2 Å was identified as sepiolite by *in situ* qualitative and quantitative EDS coupled to high resolution TEM (see Supplementary Figure S2). Study of thin sections with petrographic microscopy (results not shown) revealed dense morphologies with highly compacted large lenticular gypsum crystals in the surface layer (up to 2 mm deep) and in the bottom layer (2–3 mm) of the gypsum deposit. The appearance of these compact layers was indicative of gypsum dissolution and recrystallization processes. The interior of the sample consisted of microcrystalline (50 μm long) lenticular gypsum crystals crossed by dense horizontal bands (0.3–1 mm-wide) of verticalized sub-prismatic gypsum crystals and lenticular gypsum crystals. Sequences of vertically-elongated pores (< 2 mm-wide) and gypsum “columns” (0.1–1 mm-wide) were also observed within the interior of the gypsum deposits. These columns were formed by anhedral gypsum crystals and presented evident dissolution features. These petrological observations were consistent with porosity analyses performed with the MIP technique. MIP data showed a total porosity for connected pores (for a 0.0036 μm to 190.58 μm range) of 29.9% (*n* = 4, *SD* = 3.9) for the surface layer, 56% (*SD* = 10.1) for the rock interior, and 25.6% (*SD* = 3.8) for the bottom layer of the gypsum. Pore size distribution curves revealed larger pore diameters, between 0.1 and 190.58 μm, for the gypsum interior than for the surface and bottom layers (Figures 3A–A″). The total intrusion volume of mercury was about 0.08 ml g^−1^ for the surface and bottom layers and 0.31 ml g^−1^ for interior of the gypsum deposit. Together, these data support a significantly higher porosity for the interior than for the external layers of the gypcrete.

The transmitted PAR value within the gypsum colonization zone at a depth of 2–5 mm, we measured was 0.1–1% of that of the incident PAR light (Figure 3B). Using 2700 μmol photons m^−2^ s^−1^ as the incident PAR value for a sunny day we calculated an absolute PPFR value between 0.2 and 30 μmol photons m^−2^ s^−1^ within the cryptoendolithic colonization zone of the gypsum deposit. No UVA or UVB radiation was detected across a 1.5 mm-thick gypsum layer.

### Characterization of endolithic microbial communities

#### Characterization of endolithic microbial communities by microscopy

**Figure 4:**
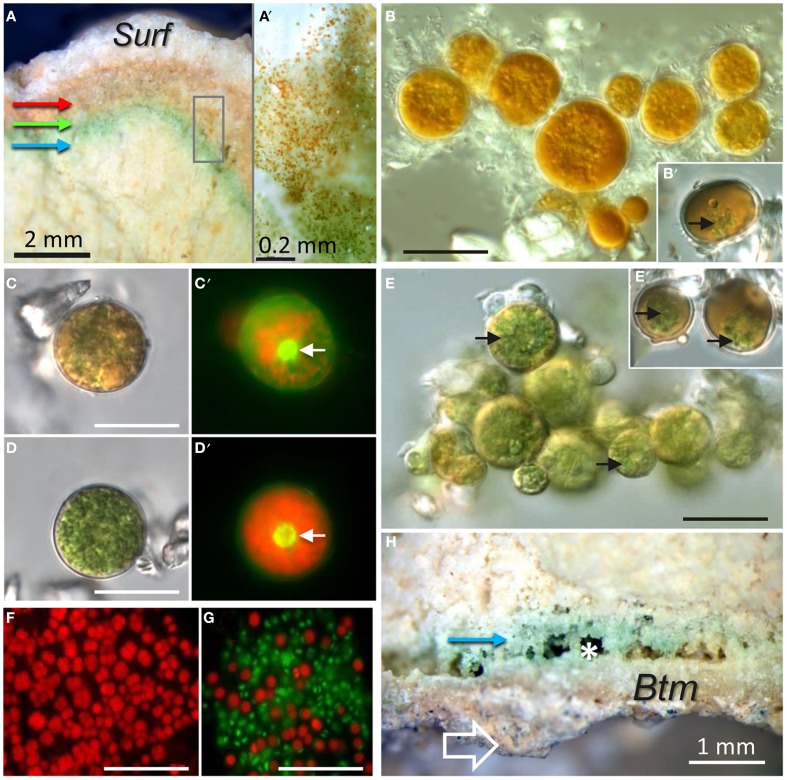
**Cross-sections of gypsum layered microbial endolithic colonization. (A)** Stereoscopic microscopy view with details in **(A**′**)** of the orange-to-green cryptoendolithic colonization layer close to the gypsum dense surface (*Surf*); red, green, and blue arrows point to orange algae, green algae, and cyanobacteria colonization zones respectively. **(B)** DIC image of orange mature algae and **(B**′**)** DIC image insert where some relicts of loose chloroplasts within the mature alga can be distinguish. **(C)** DIC and **(C**′**)** FM image of orange algae with dispersed chloroplasts [red signal in **(C**′**)**] surrounding the alga nucleus [arrow in **(C**′**)**]. **(D,E)** DIC images and **(D**′**)** FM image of green algal cells with uniformly distributed dense chloroplasts in the whole cytoplasm [red signal in **(D**′**)**; arrow in **(D**′**)** point to nucleus and arrow in **(E)** point to chloroplasts; **(E**′**)** DIC image of green cells in the first step of the carotenogenesis process with less dense chloroplasts (arrows). **(F)** MF image of cryptoendolithic cyanobacteria with red autofluorescence signal. **(G)** FM image of hypoendolithic cyanobacteria; autofluorescence red signal that potentially belong to vital cells and green signal corresponding to cells with degraded photosynthetic pigments. **(H)** Stereoscopic microscopy view of hypoendolithic cyanobacteria colonization zone (blue arrow) within gypsum close to the bottom (arrow) of the rock. Note the dense structure of the bottom (*Btm*) layer and small cave-shaped pores in the hypoendolithic habitat (asterisk). Scale bars = 20 μm, except **(A,A′,H)**.

In field fracturing of gypsum deposits revealed an abundant microbial colonization, which was clearly visible as a thick 2–6 mm orange-green layer beneath the gypcrete surface (Figure 4A). Closer examination of this cryptoendolithic habitat shows the presence of orange-colored algae cells close to the surface and green algae cells just below the orange layer of cells. This transition from green to orange cells was gradual as shown in Figure 4A′. DIC microscopy of orange mature algae showed a cytoplasm filled with orange-colored lipids and small loose chloroplasts surrounding the nucleus (Figures 4B′,C,C′). Chloroplasts within the green algae cells were denser and found throughout the cytoplasm (Figures 4D,D′,E). Green algae aggregate showed some cells with a lower density of chloroplast and starting to take orange color (Figures 4E,E′). In some places, it was also possible to observe cyanobacteria aggregates just beneath the algae layer (Figure 4F). Cyanobacteria aggregates were also found within the colonization zone at the bottom of the gypsum deposits (Figure 4G). This hypoendolithic habitat displayed a dense bottom layer and micro cave-shaped pores within the colonization zone (Figure 4H). Fluorescence microscopy revealed potentially viable cyanobacteria cells and cells with degraded photosynthetic pigments (Roldán et al., 2014) within this hypoendolithic habitat (Figure 4G).

**Figure 5:**
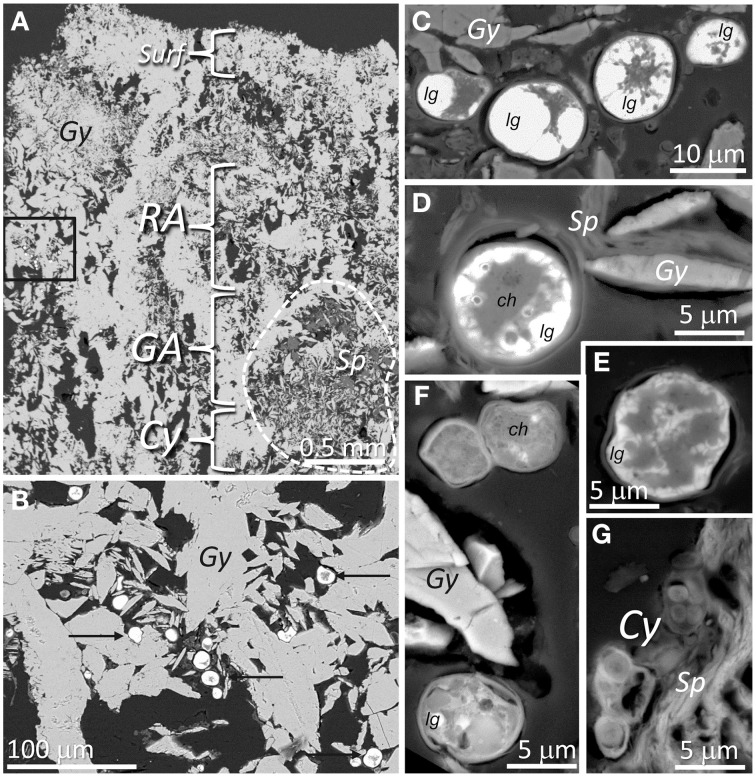
**SEM-BSE images of the gypcrete top zone with cryptoendolithic microbial habitat. (A)** Image shows surface (*Surf*) dense layer, orange-color algae colonization zone (*RA*), green algae (*GA*) colonization zone, and cyanobacteria (*Cy*) colonization zone close to sepiolite (*Sp*) nodules; square mark area shown in detail on figure **(B)**, where black arrows point to alga cells between lenticular gypsum (*Gy*) crystals. **(C–F)** *In situ* images showing algal cells among gypsum (*Gy*) crystals from higher to lower position within the colonization zone with clearly distinguishable ultrastructural elements such as lipoidal globules (*lg*) and chloroplasts (*ch*). **(G)** Image of cyanobacteria cells (*Cy*) in direct contact with sepiolite (*Sp*).

The cryptoendolithic habitat appears beneath the 0.5–1 mm-thin hardened surface layer with dense large lenticular gypsum crystals, as shown by the SEM-BSE technique (Figure 5A). In many places, large aggregates (0.5–1 mm) of clay mineral identified as sepiolite (SEM-BSE-EDS results not shown) were also observed (dash outline in Figure 5A). Algal cells were observed between smaller gypsum crystals, just beneath the hardened surface layer. A detailed view of these algae with a cytoplasm filled by lipoidal globules is shown in Figure 5B. Algal cells found deeper in the cryptoendolithic zone contained fewer lipoidal globules and their cytoplasm was mostly filled by chloroplast structures (Figures 5C–F). Cyanobacteria cells were mostly found close to sepiolite aggregates (Figure 5G) beneath the green algae layer.

**Figure 6:**
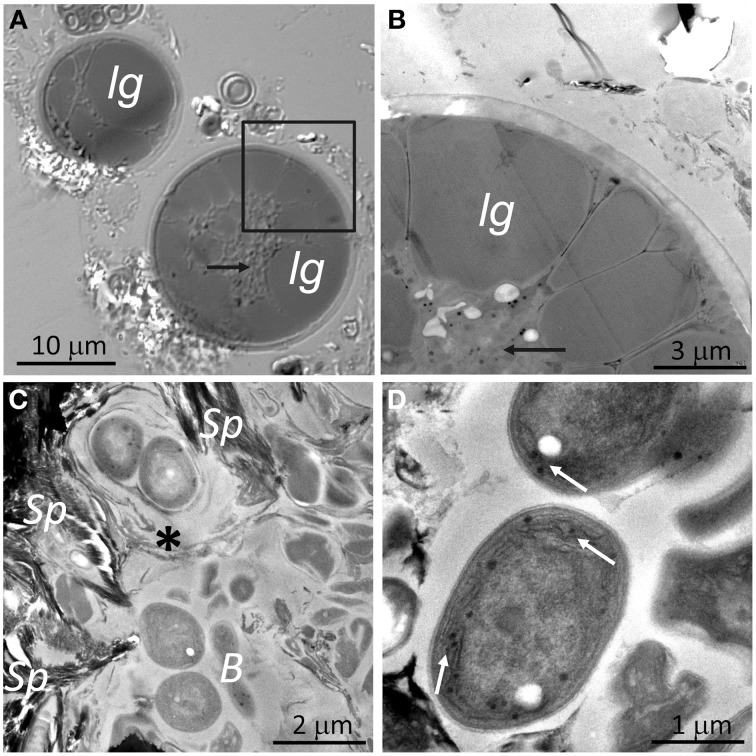
**DIC and TEM images of endolithic cells: (A) DIC image and (B) TEM image from zone marked by square in (A) showing algae from the orange zone with lipoidal globules (*lg*) within the cytoplasm (black arrows). (C)** Image showing cyanobacteria from the cryptoendolithic zone surrounded by sheaths of polysaccharides (asterisk) with heterotrophic bacteria (*B*) and close to sepiolite floccules (*Sp*). **(D)** Cyanobacteria from the hypoendolithic habitat with thylakoids (arrows).

Similarly, to DIC microscopy analyses (Figures 4B, 6A), visualization of algae from the orange zone with TEM showed abundant lipoidal globules occupying almost the entire cell (Figure 6B). These algae displayed a thick (about 0.5 μm) cell wall. TEM of algae subjacent zone revealed cyanobacteria cells surrounded by electron dense structures, forming concentric sheaths of polysaccharides and abundant extracellular material embedding heterotrophic bacteria (Figure 6C). Our TEM microscopic observations confirmed that the micromorphology of the gypsum cyanobacteria, in the top layer of the substrate, is similar to that of members of the *Chroococcidiopsis* genus (Figure 6C). Note that cyanobacteria from the cryptoendolithic habitat were always observed in close contact with floccules of sepiolite (Figure 6C). The mineralogical nature of this clay mineral was identified by *in situ* qualitative and quantitative EDS coupled to high resolution TEM (Supplementary Figure S2). Figure 6D shows *Chroococcales* cyanobacteria from the hypoendolithic habitat.

**Figure 7:**
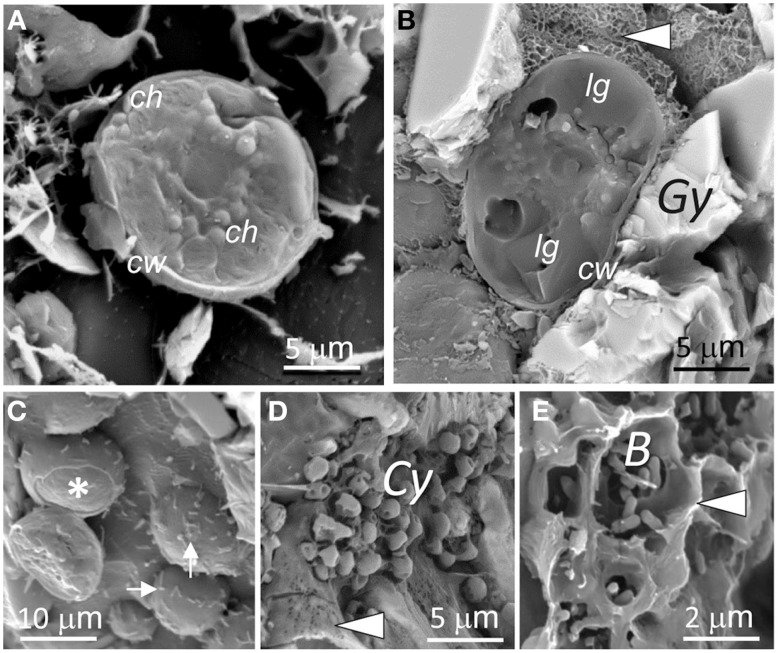
**LT-SEM images of endolithic microorganisms and extracellular polysaccharides. (A–C)** Fractured algae cells with ultrastructural elements such as: cell walls (*cw*), chloroplasts (*ch*), lipoidal filling (*lg*), and gypsum particles (*Gy*); detached cell wall, asterisk; rod shaped microorganisms (potentially heterotrophic bacteria) attached to algae, white arrows; arrow head in **(B)** point to EPSs. **(D)** Image showing cyanobacteria (*Cy*) aggregates and slime EPSs (arrow head). **(E)** Image showing bacteria (*B*) evolved by capsular EPSs (arrow head).

Visualization of cryo-fixed and cryo-fractured samples was essential in observing the gypsum microbial communities *in situ* in their “natural” state after sample rehydration (Ascaso and Wierzchos, 2002). It allowed for the characterization of internal structures in algae cells and that of the extracellular polysaccharidic substances (EPSs) surrounding microbial cells. LT-SEM of fractured algal cells from the green zone showed well defined chloroplasts filling almost the entire cytoplasm (Figure 7A). In contrast, algal cells from the orange zone accumulated lipoidal globules (Figure 7B). In many cases, we observed rod shaped (1–2 μm long) microorganisms (potentially heterotrophic bacteria) attached to the cell walls of algae (Figure 7C). Beneath the algae zone, we found aggregates of cyanobacteria (Figure 7D) and an accumulation of heterotrophic bacteria (Figure 7E). EPSs with different micromorphologies surrounded all cells. The EPSs associated with algal cells has a honeycomb-like structure (Figure 7B) whereas the EPSs in close-contact to cyanobacteria had a slime-like structure (Figure 7D). Both types of EPSs were loosely bound to the cells and did not take the shape of these cells. Groups of heterotrophic bacteria were surrounded by capsular-like polysaccharides (Figure 7E) that are distinguished according to their thickness and consistency (Rossi and De Philippis, 2015).

**Figure 8:**
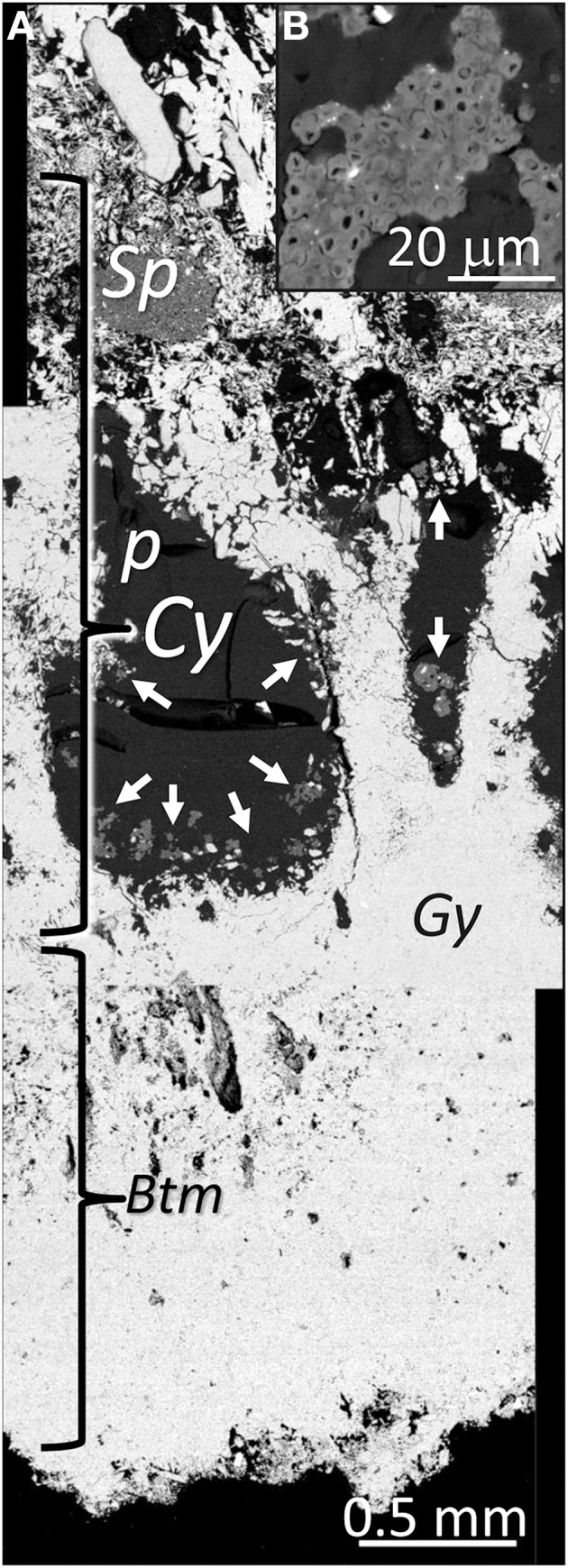
**(A)** SEM-BSE image of the bottom part of the gypcrete colonized by hypoendolithic cyanobacteria (*Cy*) forming aggregates; pore, *p*; sepiolite nodule, *Sp*; arrows point to cyanobacteria aggregates; hypoendolithic cyanobacteria colonization zone, *Cy*; bottom layer, *Btm*; gypsum, *Gy*. **(B)** Details of cyanobacteria aggregate.

The structure of the gypsum hypoendolithic habitat, which did not contain algae, was significantly different than that of the surface cryptoendolithic habitat (Figure 8). Sizeable pores (0.2–2 mm in diameter) with compacted walls were located above a compact gypsum bottom layer (Figure 8A). The structure of these pores, together with the columns formed by lenticular gypsum crystals and with the dissolution features at the edges of the anhedral gypsum crystals, similar to the “Grating Fabric” defined by Artieda (2013), revealed distinct gypsum dissolution and crystallization processes. The microbial colonization in form of cyanobacteria aggregates covered the walls of the large pores and, unlike in the cryptoendolithic habitat, did not adhere to sepiolite nodules. Many cyanobacteria cell appeared empty and were most likely dead cells (Figure 8B).

#### Micromorphological characterization of algae

**Figure 9:**
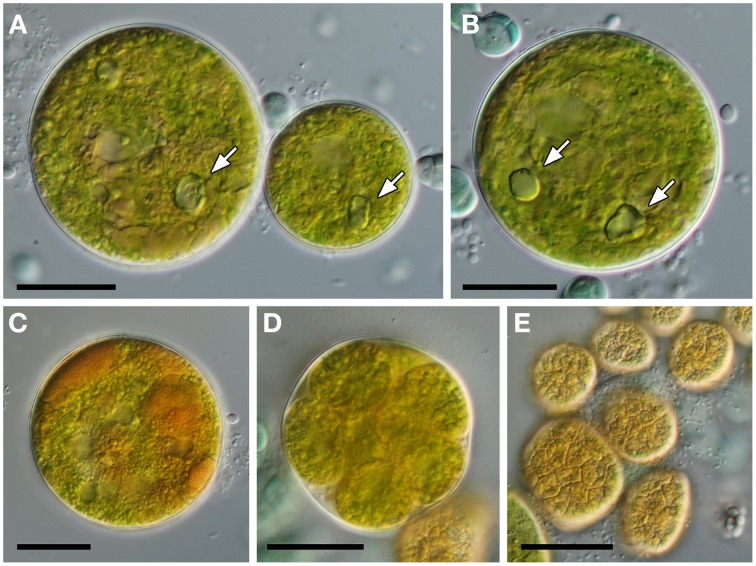
**Morphology of cultivated green alga. (A)** Size variability of vegetative cells. **(B)** Mature cell with a complex chloroplast and with several pyrenoids marked by arrows (in **A**,**B**). **(C)** Accumulation of orange color pigments. **(D)** Mature autosporangium. **(E)** Irregularly shaped autospores shortly after their liberation. Scale bars = 20 μm.

Growth of algal cells was only observed with solid BG-11 mineral medium. The growth was very slow and the great majority of algal cells died within a few weeks of their cultivation. The cultivated cells were globular, uninuclear, 28–50 μm in diameter and with smooth cell walls (Figure 9A). The cells possessed a reticulated chloroplast with a complex structure and often with several off-center pyrenoids (Figure 9B). Mature cells accumulated large quantities of orange-colored pigments (Figure 9C). Asexual reproduction occurred via 12–16 autospores, liberated by gelatinization of the maternal cell wall (Figure 9D). The autospores had irregular shape and size of 14–20 μm in diameter. They possessed a chloroplast divided into several polygonal pieces lacking pyrenoids (Figure 9E).

#### Molecular characterization of endolithic microbial communities

**Figure 10:**
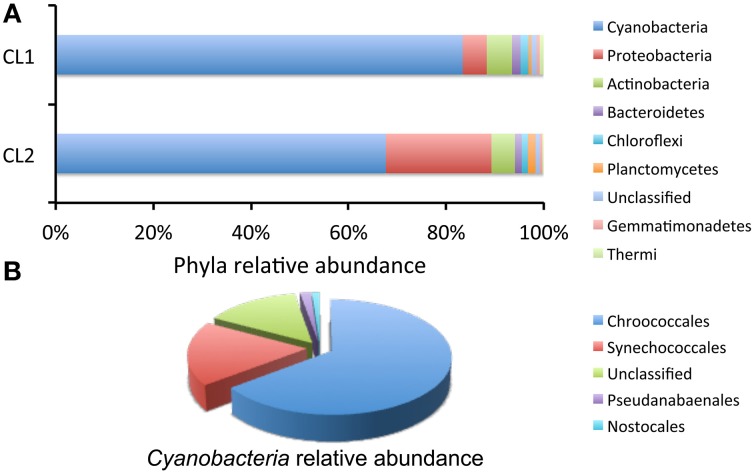
**Relative abundance of major taxonomic groups (>0.1% abundance) in the Atacama Desert gypsum community based on 16S rRNA gene sequences. (A)** Relative abundance of major phyla from 2 samples (CL1 and CL2). Datasets were of equal size and subsampled at 1662 sequence reads. **(B)** Relative abundance of Cyanobacteria taxa in combined samples.

Taxonomic assignment revealed that the dominant phyla in both samples were *Cyanobacteria*, representing between 67 and 83% of all the sequence reads, and *Proteobacteria*, and *Actinobacteria* (Figure 10A). The only other photosynthetic phylum, in addition to *Cyanobacteria*, was that of *Chloroflexi* with 1.2–1.6% of sequence reads (Figure 10A). All diversity metrics, at a maximum sequencing depth of 1662 sequences per sample, revealed a higher diversity in sample CL1, with 260 OTUs at the 0.03% cutoff, when compared with sample CL2, with 167 OTUs (see Supplementary Table S2). Across the two samples, cyanobacteria included members of the *Chroococcales, Synechococcales, Pseudanabaenales*, and *Nostocales*; the most numerous were *Chroococcales* (64%) (mostly of the *Chroococcidiopsis* genus), followed by *Synechococcales* (18%), and unclassified *Cyanobacteria* (14%) (Figure 10B). No chloroplast sequences from algae were detected indicating a considerable phylogenetic distance away from the *Cyanobacteria* or potential primer mismatches with the plastid's 16S rRNA genes. This was not the case in a previous study were we obtained amplicons from the 16S rRNA gene of the chloroplast from an algae related to the *Dolichomastix* genera (*Mamiellaceae* family) with this same primer set used in this study (Robinson et al., 2015). Despite multiple attempts at PCR amplification, using a range of algal primers, with and without nested primers, we were unable to obtain the 18S rRNA gene sequence of the alga that was visualized by microscopy and analyzed by Raman spectroscopy. Future studies, using metagenome data from the gypsum community, together with our cultivation effort, might provide the genetic characterization of this alga.

The most abundant OTUs from each of the two gypsum datasets were used to build a phylogenetic tree. Most sequences belong to *Cyanobacteria* with closest relatives from arid deserts around the world and from members of the *Chroococcidiopsis* genus (Supplementary Figure S3). Only two OTUs were from *Alphaproteobacteria*, also associated with reference sequences from extreme environments (Supplementary Figure S3).

### Raman spectroscopy of endolithic microbial communities

**Figure 11:**
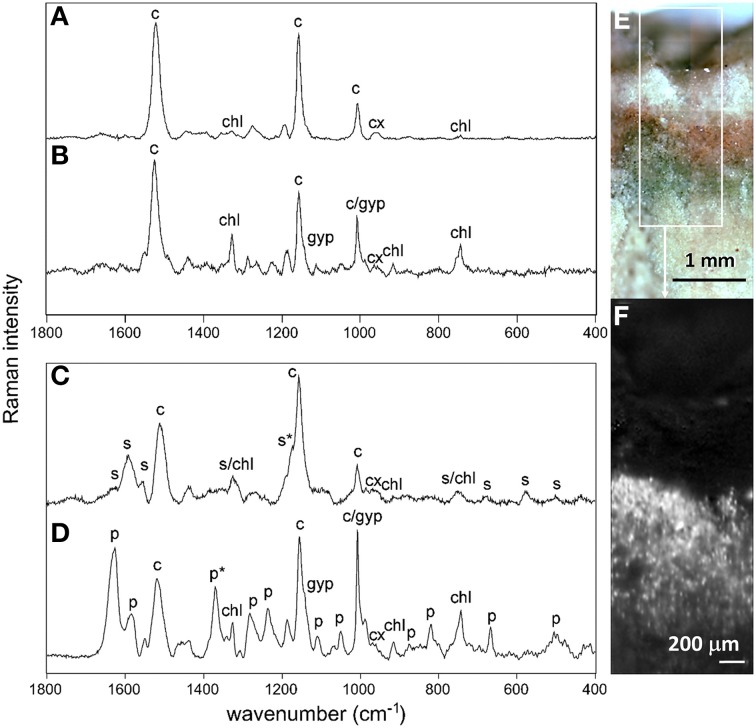
**Examples of Raman spectra of orange algal cell (A), green algal cell (B) and cyanobacterial cells from the hypoendolithic habitat containing scytonemin (C) and without scytonemin (D)**. Spectra were obtained by the 785 nm excitation source; c, carotenoid; cx, carotenoid-xanthophyll; chl, chlorophyll; p, phycobiliproteins; s, scytonemin; gyp, gypsum. Asterisks (^*^) indicate important corroborative bands for discrimination between scytonemin and phycobiliproteins. **(E)**, microscopy image of cryptoendolithic habitat colonized by orange and green algae; **(F)** Raman distribution map of carotenoid (white spots) of the marked square in **(E)**. For examples of Raman spectra extracted directly from the image see Supplementary Figure S5.

We obtained Raman spectra for both the cryptoendolithic and hypoendolithic microbial habitats within the gypsum sample (Figures 11A–D). Micro-Raman spot analysis of orange (Figure 11A) and green (Figure 11B) cells from the cryptoendolithic zone revealed spectral records dominated by carotenoid and chlorophyll signals. No phycobiliproteins were detected suggesting that both spectra belong to algal cells. The spectra showed slight differences in wavenumber position of the carotenoid bands between the green and orange cells, with the ν_1_(C = C) band position at 1521 and 1519 cm^−1^ (orange cells at 785 and 532 nm) and 1524 and 1518 cm^−1^ (green cells at 785 and 532 nm). These differences were reproducible for other areas of the substrate and were interpreted as a result of selective resonance Raman effect (see Supplementary Section S8.4 section). The Raman distribution map of carotenoids in algae from the cryptoendolithic habitat displayed a higher intensity signal just beneath the gypsum surface and a lower intensity signal close to green algae zone (Figure 11F). The method of Raman imaging proved to be extremely powerful for mapping the spatial distribution of strong Raman scatters in geobiological samples (Vítek et al., 2014). Raman signals for cyanobacteria were clearly recognized in the gypsum hypoendolithic habitat. In particular for scytonemin (Figure 11C), a UV-screening pigment characterized by the corroborative Raman bands at 1595, 1554 cm^−1^, and 1173 cm^−1^ (Edwards et al., 2000). The feature around 1322 cm^−1^ is the combination of scytonemin and chlorophyll signals. While brown-green cell aggregates produced a scytonemin signal, no scytonemin was detected within the neighboring blue-green cells (Figure 11D). Here, Raman features of carotenoids (at 1518 and 1156 cm^−1^ and Supplementary Figure S6 for Raman identification of carotenoids) and chlorophyll (1325, 915, and 743 cm^−1^) were detected. Additionally, strong bands for phycobiliproteins also revealed the presence of cyanobacteria. These accessory light harvesting pigments were characterized by the bands at 1627, 1582, 1370, 1283, 1237, 1108, 1050, 874, 820, 667, and 505 cm^−1^ (Figure 11D).

### Characterization of metabolite pigments by spectrophotometry

**Figure 12:**
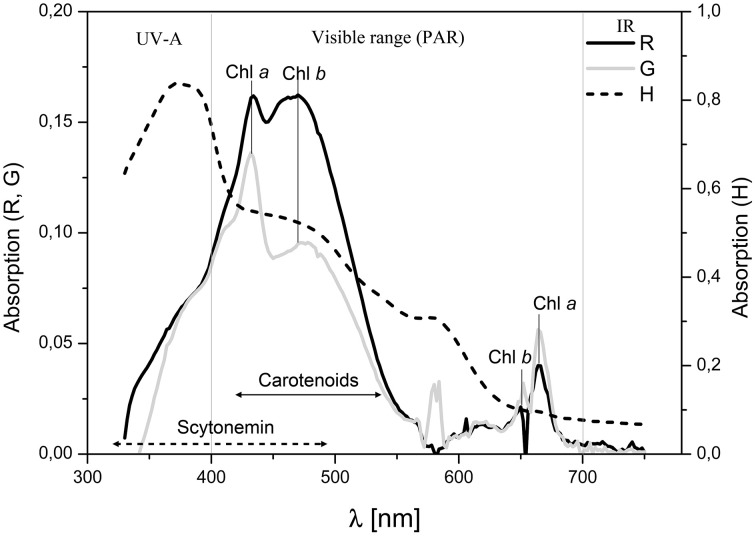
**Absorption spectra of acetone pigment extracts in the range of 320–750 nm from different endolithic colonization zones: black line, extract from orange algal zone (R), gray line, extract from green algal zone (G) and dashed line, extract from hypoendolithic zone (H)**. The characteristic peaks of chlorophyll *a* (663 nm), chlorophyll *b* (647 nm) are observed. Carotenoids and scytonemin absorption ranges are indicated by double arrowed lines.

The absorbance spectrum of pigment extracts obtained from different endolithic colonization zones are shown in Figure 12. Extracts from the orange algal cryptoendolithic zone showed high absorption values for the carotenoids absorption range. In contrast, extracts from the green cryptoendolithic colonization zone had lower absorption peaks for carotenoids and a higher chlorophyll absorption peak at 663 nm. This may indicate that the green zone also contains cyanobacteria together with green algae of lower carotenoid content. Extracts from the hypoendolithic zone showed strong absorption in the range of UVA corresponding to scytonemin absorption peaks at 315–400 nm (Proteau et al., 1993). Semiquantitative determination, based on the analysis of absorbance spectrum from extracted pigments, showed that the gypsum orange zone had twice the content of carotenoids and less of both chlorophylls *a* and *b* than the green zone (Supplementary Table S2).

## Discussion

### How extreme is the environment at the colonized gypsum site?

Most of the Atacama Desert is characterized by persistent extremely dry conditions and scarce precipitations in its hyperarid area (Houston and Hartley, 2003; Hartley et al., 2005; Houston, 2006a). Based on an evapotranspiration rate of 2920 mm y^−1^ for the nearby Salar de Atacama basin (Houston, 2006a), and yearly precipitations of 27.1 mm (this study), the aridity index for the Cordon de Lila sampling site is as low as 0.0093, presenting exceptionally hyperarid conditions. The extreme aridity of this location is also expressed by long time-spans between scarce precipitations and also extremely low relative humidity. The mean annual RH value of 16.5% at Cordon de Lila is even lower than the value of 17.3% reported by Azua-Bustos et al. (2015) and recently reported as the driest site of the hyperarid Atacama Desert. Chile's northern Atacama Desert is also known as one of the places on Earth with the highest surface UV radiation (Cordero et al., 2014). The most extreme values for solar total and UV irradiances (1528 Wm^−2^) were reported for “Puna de Atacama” by Piacentini et al. (2003) and by Cabrol et al. (2014) (43.3 UV index), in a region of high altitude, with mostly cloudless conditions, relatively low total ozone column, and extremely low RH values. Recently, Rondanelli et al. (2015) show that the location of Earth's surface solar radiation maximum is in the Atacama Desert in the region between 24° and 25°S along 69°W, over what is known as the Domeyko Cordillera. While solar radiation is essential for phototrophic life, extremely high levels of UV radiation is harmful to cellular components (Jeffrey et al., 1996; Phoenix et al., 2006), and excessive PAR results in photo-inhibition by damaging the reaction center of photosystem II in photosynthetic microorganisms (Häder, 1986). By combining both hyperaridity and extreme solar irradiance, the Cordon de Lila site with endolithically colonized gypsum studied here holds world records for environmental extremes, leading to the virtual absence of any life form on its rocks and soil surfaces.

### How extreme is the environment within the gypcrete endolithic microhabitat?

The effectiveness of gypsum in attenuating solar irradiance has been evaluated by several studies. Amaral et al. (2007) reported UV transmission of less than 20% through a 0.5 mm-thick gypsum layer. Effective attenuation of light, leading to transmission of 0.23% to 0.5% (from 300 to 680 nm) of incident light through a 0.9 mm thick Arctic gypsum crust, was reported by Cockell et al. (2010). The transmission of only 0.005% of the UVB and 0.05% of UVA, and 1% of PAR, were reported in a 1.2 mm thick Antarctic endolithically colonized gypsum by Hughes and Lawley (2003). In contrast, a 1 mm thickness of Arctic selenite exhibited only 25% reduction in ambient UV exposure (Parnell et al., 2004), underlying the importance of the micromorphology of gypsum crystals and the compactness of the gypsum layer in solar irradiance attenuation. Our data for the gypsum cryptoendolithic colonization zone, showing no UV transmission and 0.1–1% of PAR transmission, are consistent with previously reported studies. However, when taking into account the extreme solar irradiance at the sampling site and the small zenith angle, between 5° and 25°, experienced at the site when rain water is potentially available (July-March), it is likely that the cumulative dose of PAR within the endolithic habitat is extremely high.

Water is an essential element for the successful colonization of the gypsum deposits. The presence of cracks, fractures and pores in the gypsum substrate allow rainfall water and nutrients to penetrate into the gypcrete. Using EC measurements, we estimated that liquid water was present at the rock surface for less than 10 days per year. However, within the gypsum interior, the moisture regime could be significantly higher. We have observed compact structures and low porosity at the surface and bottom layers of the gypsum deposits that might significantly decrease water evaporation rates. We also identified gypsum crystal dissolution and crystallization patterns mostly at the bottom layer, indicating the presence of liquid water, within the rock, for some periods of time. These observations lead to what we call the “eggshell” shield effect in which the gypsum interior is protected against rapid water evaporation rates. Moreover, the water can be efficiently trapped and retained inside the gypsum deposits by the sepiolite nodules randomly distributed in the substrate. Sepiolite is a fibrous sheet of magnesium silicate clay with unique physicochemical and rheological properties due to the presence of zeolitic channels in its structure (Leguey et al., 2014). Its nano-porosity induces efficient water absorption and retention up to 0.3 g g^−1^ dry weight (Caturla et al., 1999). We suggest that the sepiolite water retention capacity might increase significantly the fraction of water available to endolithic microorganisms.

### Sub-micro endolithic habitats and their colonizers

**Figure 13:**
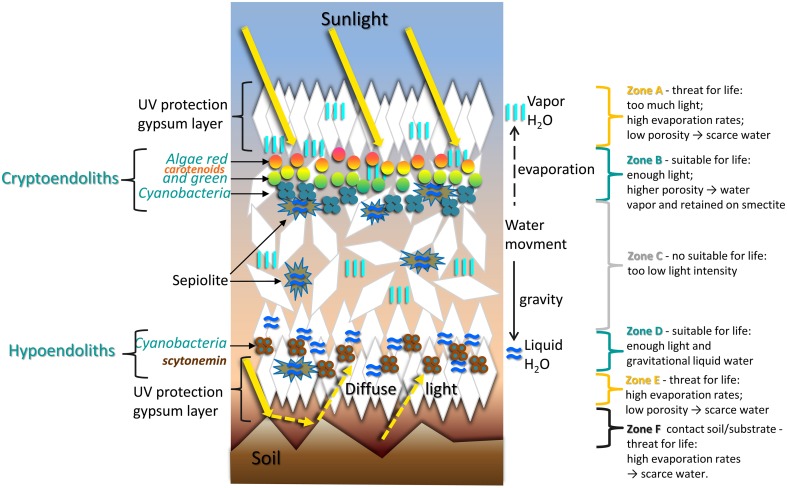
**Schematic representation of sub-micro environments with corresponding cryptoendolithic and hypoendolithic habitats within gypsum deposits from the Atacama Desert**.

Our molecular characterization of the gypsum endolithic microbial community revealed a community dominated by cyanobacteria, mostly of the *Chroococcidiopsis* genus. The difference in community structure and composition we observed between samples suggest a high level of heterogeneity in the substrate's architecture, providing sub-micro habitats for colonizing microorganisms. A model of the gypsum deposit “architecture” with its cryptoendolithic and hypoendolithic habitats, gypsum layers, and sepiolite nodules, together with the role played by these structures and by the prevalence of gaseous or liquid water is illustrated in Figure 13.

In this model, we hypothesize that the water prevalence states and therefore the moisture regime inside the gypcrete is dependent on the depth, fabric, and others accompany to gypsum minerals. Following rainfall, water fills the fissures of the rock, forming percolation pipes and conducting the water toward the gypsum's bottom layer. There, the water, or rather a calcium sulfate solution, accumulates for longer times, filling the large pores or “micro-caves” we observed. The processes of gypsum dissolution-recrystallization and water evaporation convert the bottom layer of the gypcrete into a compact, thick layer with low permeability and potentially higher UV transmission rates (Parnell et al., 2004). Liquid water is also retained within sepiolite nodules and pore spaces inside the gypcrete, providing sub-micro environments with high moisture until the gypsum is completely dried out.

The first adaptation strategy for microorganisms in hyperarid deserts is the avoidance of extreme external conditions by escaping into the endolithic habitat (Karsten and Holzinger, 2014). The second step is to follow the water, which is essential for the metabolic activity of the primary producers in the community, followed by the level of PAR. This explains the well-defined colonization patterns we observed for the algae and cyanobacteria. We propose that moisture is the key abiotic factor driving the spatial distribution of chlorophototrophs in the gypsum sub-micro habitats, providing there are sufficient PAR levels. The cryptoendolithic habitat is an area with scarce liquid water with the exception of sepiolite moisture islands and pores, with high RH values, among gypsum crystals. Our results show that this habitat is dominated by a layer of green/orange algae and a subjacent layer of cyanobacteria attached to sepiolite nodules. Cultivation of the endolithic green algae showed that the orange- and green-colored layers were formed by the same organism. This alga very probably belongs to the class *Chlorophyceae* (Chlorophyta), known to contain several desert soil algae (Lewis and Lewis, 2005). Morphologically, the gypsum alga resembles the genera *Spongiochloris, Neochloris*, and *Deasonia*. However, algae from natural gypsum cryptoendolithic habitat exhibit a distinct suite of morphological features that is unique among all described algal genera. Therefore, we conclude that the alga observed in this study belongs to a new, yet undescribed genus of *Chlorophycean* green algae.

Previous works indicated that, in arid environments, high relative humidity was enough to induce metabolic activity in algae. For example, photobionts from cryptoendolithic lichens inhabiting sandstones of the Dry Valleys of Antarctica begin to photosynthesize at *RH* = 70% (Palmer and Friedmann, 1990a) and cultured algae (*Trebouxia* sp.) are capable of photosynthesis at a RH of 80% (Palmer and Friedmann, 1990b). In contrast, endolithic cyanobacteria inhabiting sandstones in the Negev Desert require *RH* > 90% for photosynthesis (Palmer and Friedmann, 1990a). These findings may explain the close contact of cyanobacteria with sepiolite nodules within the gypsum. However, the hypoendolithic habitat in the gypsum deposits is an area with periodical occurrence of liquid water after rainfalls hence it is colonized by cyanobacteria colonies covering the walls of micro-caves. The hypoendolithic habitat was first described within the gypsum crusts collected in a less extreme (from the point of view of high night RH values) zone of the Atacama Desert (Wierzchos et al., 2011; Vítek et al., 2013). However, these authors reported the presence of algal *Trebouxia*-like cells aggregates closely associated with fungal hyphae as the main hypoendolithic colonizers of the bottom gypsum crusts, suggesting scarce liquid water within that hypoendolithic habitat.

Light penetration has been considered a primary control for the location and extent of colonization by endolithic phototrophic microorganisms (Walker and Pace, 2007). Endolithic phototrophs from Antarctica (gypsum and sandstone), and other regions around the world, were found to receive as little as 0.01% of incident light (Nienow et al., 1988; Matthes et al., 2001; Hughes and Lawley, 2003). In our study, we did not observed chlorophototrophs below 0.1% of incident PAR in the cryptoendolithic habitat. We suggest that the additional hyperaridity found at the Cordon de Lila sampling site could potential hinder the colonization of deeper substrate layers where PAR levels are below 0.1%. However, despite relatively low values of transmitted PAR, absolutely PAR values were as high as 30 μmol photons m^−2^ s^−1^, potentially inducing chlorophototrophs to produce abundant secondary metabolites such as carotenoids and scytonemin. High rates of solar irradiance increase considerably the probability of photoinhibition and photooxidative lethal damage to phototrophs (Solovchenko and Merzlyak, 2008). Photoinhibition has been defined as the inhibition of photosynthesis caused by excessive radiance; it may damage the photosynthetic apparatus, causing the (photo-) destruction of the photosynthesizing pigments (Long et al., 1994; Alves et al., 2002). It is therefore likely that the algae and cyanobacteria colonizing the gypsum endolithic habitat have evolved self-protective strategies such as the efficient production of secondary metabolites shielding them against UV and PAR excessive irradiance.

### Solar irradiation protective pigments nature and their spatial distribution

Carotenoids are the accessory pigments of the photosynthetic apparatus, which participate in both light-harvesting and photoprotective functions (Nguyen et al., 2013). Some short-chained carotenoids can potentially act directly as UV-screening pigments but are not favored by organisms, whereas ubiquitous carotenoids with nine or more conjugated double-bonds absorb more in the light visible region (Cockell and Knowland, 1999). These long-chain carotenoids are able to cope with toxic reactive oxygen species that can form during photosynthesis, or as the result of UV-irradiation, and which would destroy the biomolecules forming the photosynthetic apparatus (Asada, 1994; Telfer et al., 2008).

From our Raman spectroscopic results, we observe a clear enhancement of carotenoid/chlorophyll intensity ratio of Raman signal in the case of the orange-color algal cells compared to the green-color algal cells. All the band positions may be attributed to a standard C-40 carotenoids, with bands above 1520 cm^−1^ probably due to xanthophyll compounds such as zeaxanthin and lutein, and the band at 1514 cm^−1^ assignable to β-carotene. However, based just on the Raman spectra alone the exact and unequivocal identification of a particular carotenoid in these cells was not possible, as the ν1 band position can be identical for several carotenoids or different for the same carotenoid in different biological matter (de Oliveira et al., 2010). We interpret the differences in relative carotenoid content (increased within orange algal zone) and carotenoid composition as a response of algae to light-driven stress enhanced with the proximity to the surface. The relative intensity of chlorophyll Raman spectral features differs significantly between green and orange algal cells. The clear characteristic signals of chlorophyll were detected within the green cells using 785 nm laser for excitation, represented by the bands at 1326, 918, and 745 cm^−1^. On the other hand, spectra from the orange cells revealed none or very weak chlorophyll features. The very weak signal of chlorophyll in the orange cells was the result of decreased chloroplasts content in these cells, which was clearly confirmed by microscopy observations.

The capability to synthesize very high amounts of a complex mixture of secondary carotenoids under environmental stresses is widely spread over the green microalgae, as a defense mechanism against environmental injuries. Secondary carotenoid formation enhances the cell resistance to oxidative stress generated by unfavorable environmental conditions including excess light, UV and PAR irradiation, and nutrition stress and, therefore, confers a higher survival capacity to the cells (Hanagata and Dubinsky, 1999; Orosa et al., 2000, 2001). High light levels and salinity were reported as main stress drivers enhancing carotenogenesis in the aerial microalga *Scenedemus* sp. (*Chlorophyceae*) leading to the production of xanthophylls as fatty acid esters in the algal cells (Aburai et al., 2015). Moreover carotenoids have been identified as photoprotection agents against photoinhibition and molecular damage of the photosynthetic apparatus (Oren et al., 1995).

Our analytical results obtained by spectrophotometry and Raman spectroscopy demonstrate enhancement in carotenoids and depletion in chlorophyll photosynthetic pigment within orange cells lying close to the gypsum surface. This progressive carotenogenesis process in the case of the cryptoendolithic algae from the Atacama's gypsum deposits is driven by higher PAR light levels toward the gypsum surface, the oligotrophic environment, and the very large periods (up to 9 months) of dehydration followed by desiccation. It was also described that carotenogenesis processes might lead certain algal cells to turn from a green to an orange/red-color (e.g., “blood alga” *Haematococcus pluvialis*, snow alga *Chlamydomonas nivalis* or *Scenedesmus komareki*) due to the accumulation of large amount of carotenoids within the cell (Hanagata and Dubinsky, 1999 see also references therein).

Carotenogenesis generally accompanies larger metabolic changes and morphological modifications, i.e., progressive accumulation of lipoidal globules and/or disappearance of certain cell organelles such as chloroplasts (Hanagata and Dubinsky, 1999). A similar process has been observed in the case of the cryptoendolithic algae, where lipoidal globules that were first formed at the periphery of the cell, progressively propagated toward its inside, eventually filling most of it. The chloroplast was single and parietal in the green cells. As the cells turned orange, the chloroplast divided into several small lobes and was pushed toward the interior of the cell by the accumulating lipoidal globules. However, our knowledge of the cellular localization of secondary carotenoids and the change of cellular ultrastructure associated with their accumulation in other carotenoid-accumulating algae is limited and will be the topic of future studies.

The presence of scytonemin, a well-known UV-screening pigment, in the gypsum hypoendolithic habitat was confirmed by Raman spectroscopy and spectrophotometric analyses. Scytonemin is a typical cyanobacterial metabolite that is extracellularly located in the cyanobacteria exopolysaccharide sheath around cells and cell aggregates, as recently shown for endolithic cyanobacteria colonizing Atacama's halite (Vítek et al., 2014). Scytonemin is known to have photoprotective properties against harmful UV radiation (Garcia-Pichel and Castenholz, 1991; Cockell and Knowland, 1999; Vítek et al., 2014). Periodic desiccation of cyanobacteria in combination with high UV fluxes may further induce scytonemin biosynthesis (Fleming and Castenholz, 2007). This raises the question of how can UV irradiate hypoendolithic cyanobacteria? First, it should be mentioned that hypoendolithic colonization has only been found in these specific gypsum deposits, which were detached from the coarse grain soil underneath, suggesting that diffused solar light could illuminate the gypcrete underneath. In addition, as mentioned above, the bottom gypsum layer shows a very compact structure potentially permitting some transmission of UV (Parnell et al., 2004) from intense solar irradiance.

### EPSs and their role in reducing water stress and UV radiation

Our results show abundant presence of exopolysaccharides with different micromorphology surrounding algae, cyanobacteria, and heterotrophic bacteria single cells and colonies. EPSs are an important class of biopolymers, which play key protective and structural roles. Under extremely low water availability, large periods of dehydration, and excessive solar irradiance, the protection against water stress and UV and PAR radiation is one of the main roles of EPSs in constrained environments (Rossi and De Philippis, 2015). Although the role of EPSs in reducing water stress has not been fully clarified, they are reportedly involved in maintaining hydration because of their hydrophilic/hydrophobic characteristics (Makhalanyane et al., 2015). Hygroscopic EPSs act as a water reservoir after wetting events (Gorbushina, 2007) and as a possible mechanism for absorption and retention of water vapor, as reported for hypolithic microbial communities from the Dry Valleys of Antarctica (de los Ríos et al., 2014b). In addition, it was described that EPSs may contain UV-absorbing compounds, such as scytonemin (Vítek et al., 2014).

### Architecture of a lithic substrate

As observed with microscopy techniques, internal structural elements such as porosity, pore-size distribution, presence of large pores and cavities, light transparency, and light scattering properties, dissolution and crystallization features, and sepiolite nodules distribution varied significantly within various locations of the gypsum substrate. All together these structural, physical, chemical and mineral properties give rise to a new understanding of the features and functions relevant to the rock bioreceptive characteristics. We suggest using the term of “rock architecture” instead of “rock structure” to emphasize the functional role of the rock interior. As such, this new concept of the *architecture of a lithic substrate* encompasses all the internal structures of a rock that are essential as a habitat for microbial life. It is about perceiving the rock interior from the existence of porous spaces of different sizes and shapes, interconnected or not; the solid structures that divide and support these spaces, and the minerals and salts that can be transformed. All these components and elements are interrelated and influence one another, thus fulfilling a requisite: they shape a suitable architecture to hold microbial life. The architecture of habitable rocks provides resources (water, light, and nutrients, above all) and guarantees effective protection from excessive evapotranspiration, thus assuring efficient gas exchange. It also provides a long-term stable environment for life. Considering the architecture of a rock provides an integrated view of its potential habitability for endolithic microbial communities. All porous rocks have a structure, yet very few show such a suitable architecture for endolithic microbial colonization, even under extreme environmental conditions, as the Atacama's gypsum do.

## Concluding remarks

Here we report that architectural features of translucent gypsum rocks with sepiolite inclusions provide increased water availability and attenuation of harmful UV and PAR radiation to endolithic microbial communities. Intense PAR radiation induced chlorophototrophic microorganisms to form a unique layering of algae and cyanobacteria within the cryptoendolithic habitat and the synthesis and accumulation of carotenoids in the upper algae zone. Scytonemin pigment is produced by the hypoendolithic cyanobacteria to avoid photoinhibition and photooxidative damages. We found that the spatial distribution of photosynthetic pigments within the gypsum rock such as carotenoids, chlorophylls and phycobiliproteins is linked to different colonization zones and types of microorganisms. The highest concentration of carotenoids was detected in orange-colored algal cells just beneath the gypsum surface, suggesting that the green microalgae use this shielding as a survival strategy against the intensive solar irradiance. This in turn allows green microalgae to colonize a slightly deeper layer in the rock until maturation and transformation into orange-colored algae with high carotenoid content. The same carotenoid-like “umbrella” is also protecting cyanobacteria cells underneath the layer of green microalgae cells. The detection of scytonemin in the hypoendolithic habitat colonized by cyanobacteria can be interpreted as another adaptation strategy against excess of UV and PAR in this unique conformation. The highly specialized microbial assemblages we describe here can be use as model systems to further our understanding of photoprotective mechanisms under extremely unfavorable conditions.

### Conflict of interest statement

The authors declare that the research was conducted in the absence of any commercial or financial relationships that could be construed as a potential conflict of interest.
