# Supplementary material for: Adaptation strategies of endolithic chlorophototrophs to survive the hyperarid and extreme solar radiation environment of the Atacama Desert
Source: Front Microbiol. 2015 Sep 10;6:934. doi: 10.3389/fmicb.2015.00934 (PMC4564735; doi:10.3389/fmicb.2015.00934)
Supplement: Supplementary file 1 [file DataSheet1.DOCX]

***Supplementary Material***

**Adaptation strategies of endolithic chlorophototrophs to survive the hyper-arid and extreme solar radiation environment of the Atacama Desert**

**Jacek Wierzchos^1^*, Jocelyne DiRuggiero^2^, Petr Vítek^3,4^, Octavio Artieda^5^, Virginia Souza-Egipsy^6^, Pavel Škaloud^7^, Michel Tisza^2^, Alfonso F. Davila^8^, Carlos Vílchez^9^, Inés Garbayo^9^ and Carmen Ascaso^1^**

^1^Museo Nacional de Ciencias Naturales, MNCN - CSIC, Madrid, Spain

^2^Biology Department, The Johns Hopkins University, Baltimore, MD, USA

^3^Laboratory of Ecological Plant Physiology, Global Change Research Centre, Bělidla, Brno, CZ. Rep.

^4^Institute of Geochemistry, Mineralogy and Mineral Resources, Charles University, Prague, CZ. Rep.

^5^Departamento Biología Vegetal, Ecología y Ciencias de la Tierra, Universidad de Extremadura, Plasencia, Spain

^6^Instituto de Ciencias Agrarias, CSIC, Madrid, Spain

^7^Department of Botany, Charles University, Prague, CZ. Rep.

^8^Carl Sagan Center, SETI, Mountain View, CA, USA

^9^Facultad de Ciencias Experimentales, Universidad de Huelva, Huelva, Spain

*** Correspondence:** Jacek Wierzchos, Dept. Biogeoquímica y Ecología Microbiana, Museo Nacional de Ciencias Naturales MNCN – CSIC, c/ Serrano 115 dpdo., 28006 Madrid, Spain.

[j.wierzchos@mncn.csic.es](mailto:j.wierzchos@mncn.csic.es)

**Table S1. Characterization of gypsum endolithic colonization in different climates**

**S1. Electrical Conductivity sensor description**

**S2. X-ray diffraction analyses**

**S3. Measurements of light conditions within the gypsum deposits**

**S4. Fluorescence Microscopy**

**S5. Transmission Electron Microscopy**

**S6. Raman spectroscopy analyses**

**S7. Spectrophotometric measurements – Table S2.**

**S8. Analytical results**

**S8.1. Microclimate parameters evolution – Fig. S1.**

**S8.2. Sepiolite EDS analysis – Fig. S2.**

**S8.3. Molecular characterization of endoliths – Table S3, Fig. S3**

**S8.4. Raman spectroscopy analyses - Fig. S4**

**S8.4.1. Raman identification of carotenoids - Fig. S5**

**S9. References**

**
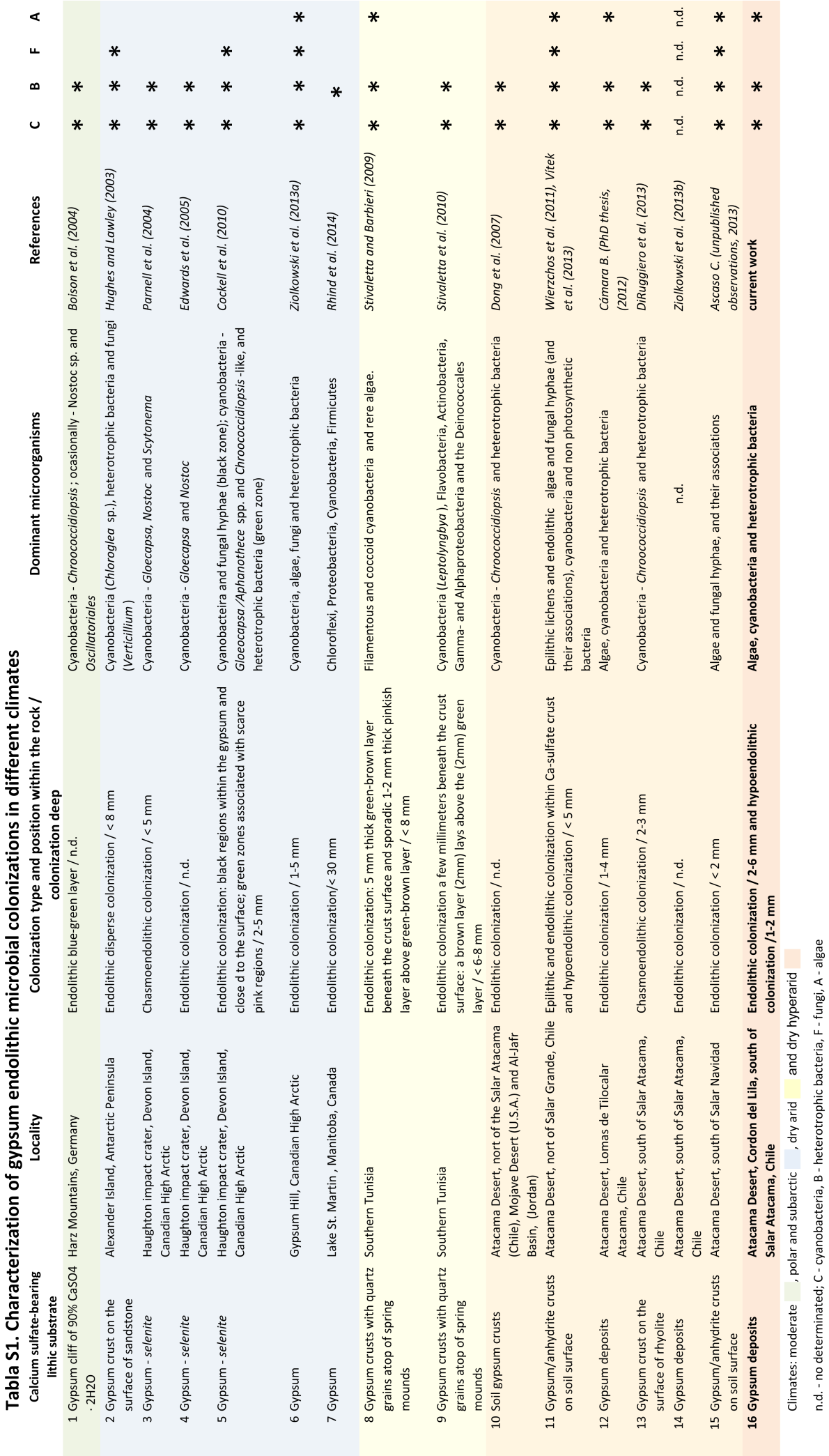
**

**Table S1. Characterization of gypsum endolithic colonization in different climates.** We do not include references describing microbial colonization in wet environment, such as salt evaporation ponds and/or sabkha, because environmental conditions at these habitats are quite different from dry sulfate-bearing rocks and soil sulfate-rich deposits from desert zones.

**S1. Electrical Conductivity sensor description**

The presence of liquid water on the gypsum duricrust surface was determined using an electrical conductivity (EC) sensor composed of a 12-bit voltage input adapter (HOBO S-VIA-CM14) interfaced with a sensor providing VDC signals and acting as a smart sensor with the HOBO data logger. This input adapter provided a trigger source voltage signal (sensor trigger source: voltage 2.5 V, maximum current: 1 mA) that powered external sensors and an open collector trigger. As external sensors, we used platinum wires (diameter 0.8 mm) connected to the trigger source input adapter gate. Two of these 10-mm long Pt wires were positioned in parallel 10 mm apart, and tightly attached to the gypsum duricrust surface by their ends with epoxy resin. The occurrence of liquid water on the rock surface was recorded as a rise in electrical conductivity (EC) over a baseline voltage of 0.0006 V (cited in the text and figures as EC > 0) up to 2.5 V, assuming that even the smallest quantity of liquid water in the system would produce a voltage increase from the baseline value. We assumed that readings from these EC sensors were indicative of wet/dry conditions on the rock surface as reported by (Omelon et al., 2006; DiRuggiero et al., 2012; Wierzchos et al., 2012; Robinson et al., 2013; Wierzchos et al., 2013).

**S2. X-ray diffraction analyses**

We used X-ray powder diffraction (XRD) on a Philips X’Pert diffractometer with graphite-monochromated CuKα radiation to identify the mineralogical composition of the gypsum duricrusts samples (five samples per site). Three kinds of subsamples were analysed: (A) samples from the surface dense crust of the gypsum located just above the colonization zone, (B) samples from interior of the gypsum duricrust and within the colonization zone, and (C) samples from to the bottom dense crust covering from the bottom hypoendolithic colonization zone. The XRD patterns were obtained from random powder mounts. For qualitative analysis of the crystalline phases present in the sample, the Power Diffraction File (PDF-2, 1999) of the International Centre for Diffraction Data (ICDD) was used. A semi-quantitative analysis of these phases was performed using the normalized RIR method (Chung, 1974) and the reference intensity ratio (RIR) values for each phase given by the powder diffraction database (International Centre for Diffraction Data, ICDD).

**S3**. **Measurements of light conditions within the gypsum deposits**

The Spherical Micro Quantum Sensor (US-SQS, Walz, Effeltrich, Germany) connected to data logger (ULM-500, Walz, Effeltrich, Germany) is designed to measure photosynthetically active radiation (PAR) between 400 and 700 nm. More specifically, this sensor measures the photosynthetic photon fluence rate (PPFR), which is the amount of PAR hitting a sensor small sphere from all directions weighted equally and divided by the cross-sectional area of the sphere and time-interval (Braslavsky, 2007). The unit for PPFR is the same as for PAR [μmol photons m^-2^ s^-1^]. The use of small spherical sensors provides measurement of not only the transmitted light radiation but also ensures the detection of scattered light within the gypsum duricrust, revealing true light conditions available to phototrophic microorganisms. The sensor consists of a 3.7 mm in diameter highly scattering plastic sphere. The measurements were performed on three natural samples with the sensor introduced into a drilled hole (4 mm in diameter) from the bottom side of the sample (left image). We started measurements at 8-12 mm from the surface of the sample. After each reading, the sensor was taken out and hole drilled (Dremel tool) some decimals of a millimetre towards the surface. The distance between the sensor and the rock surface was calculated, the sensor reintroduced, the hole taped with black wax, and a new measurement was taken. Measurements were performed in the field between 12:00 and 13:00 with a sun PAR recorded values of 2130 to 2220 μmol photons m^-2^ s^-1^. During all measurements the gypsum surface was maintained perpendicular to the sun position and the results were expressed as a % fraction of incident PPFR.


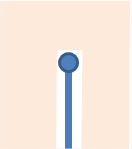


The measurement of UVA (315-400 nm) and UVB (280-315 nm) radiation were performed using a Delta OHM LP9021 UVA and UVB Irradiance Meter Cosine Corrected sensors connected to a Delta OHM DO9721 Data Logger. For this purpose five gypsum samples with distinct cryptoendolithic colonization layers were sawed and sanded (with sand paper nº 320) from the bottom up to the upper part of the colonization zone at about 1.5 mm from the rock surface. The gypsum samples were then mounted over the UVA and UVB sensors windows with sticky black foam to avoid any incoming light from the sides and the bottom. During the measurements the gypsum surface was maintained perpendicular to the sun position. Three gypsum samples without colonization zone and with a thickness of 2-4 mm were processed in the same manner.

**S4. Fluorescence Microscopy**

The fluorescence microscope was equipped with a Plan-Apo 60x ⁄ 1.4 Zeiss oil-immersion objective. Filter sets for eGFP (Zeiss Filter Set 38; Ex ⁄ Em: 450–490 ⁄ 500–550 nm) and rhodamine (Zeiss Filter Set 20; Ex ⁄ Em: 540–552 ⁄ 567–647 nm) were used for green and red signal visualization, respectively. Images were recorded using a CCD Axiocam HRc (Zeiss) camera and AxioVision 4.7 (Zeiss) software. Microbial cells were also visualized in wild field mode and differential illumination contrast (DIC) mode.

**S5. Transmission Electron Microscopy**

Fragments of material from distinct cryptoendolithic and hypoendolithic colonization zones from freshly fractured rocks were gently scraped into vials with 3% glutaraldehyde in 0.1M cacodylate buffer and incubated at 5°C for 3 hours. The cells were then washed three times in cacodylate buffer, postfixed in 1% osmium tetroxide for 5 hours before being dehydrated in a graded series of ethanol and embedded in LR White resin. Ultrathin sections were stained with lead citrate and observed with a Zeiss EM910 transmission electron at 80 kV of acceleration potential. Some of the ultrathin sections containing cyanobacteria aggregates and clay minerals were observed with high resolution TEM (Philips CM 200, Amsterdam, The Netherlands) at 200 kV of acceleration potential. The clay minerals were characterized using energy dispersive X-ray spectroscopy (EDS) microanalysis procedure using sepiolite as a standard for quantitative analysis.

**S6. Raman spectroscopy analyses**

The Raman microspectrometer was equipped with a Leica microscope with a standard 50x objective and with long-working distance objective (50x). The laser power was typically set to 3–15 mW using 785 nm excitation and 1–2 mW using 532 nm laser excitation to obtain an optimal Raman signal and simultaneously avoid any thermal alteration of the sample. Typically, 15 s scans were accumulated 1–20 times in the case of 785 nm and 0.5-2 s scans accumulated 20-40 times when a 532 nm laser was employed. The parameters depended on the signal-to-noise ratio from the particular areas.

**S7. Spectrophotometric measurements**


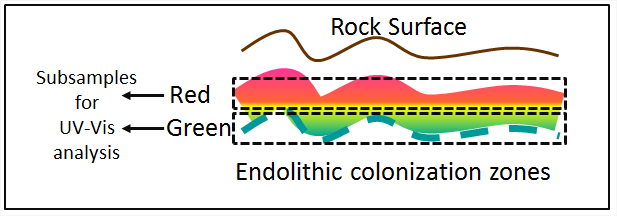
Fragments of endolithic colonization zones with adherent gypsum crystals were removed from gypsum rocks for UV-Vis spectrophotometry. Gypsum rocks were sanded (with sand paper nº 320) and polished material from areas presenting distinct signs of colonization was collected as follow for each rock: two subsamples were collected from (a) the orange zone (R) – mainly composed by orange colored algae and from (b) the green zone (G) – mainly composed by green colored algae and cyanobacteria (see diagram above). Because colonization zones do not follow a plane parallel to the rock surface, some “cross contamination” between zones was to be expected. Approximately 1.5 g of powdered rock from each zone for 4-5 different samples was collected. The material from each zone was divided into 4 subsamples, weighed (dry weight – DW), and 0.1 mL of distilled water was added to each subsample. Following overnight incubation, the samples were grinded in an ice-cold agate mortar with 0.05 g of sodium ascorbate (antioxidant) and 3-4 mL of 100% acetone (also cooled with ice). The suspension was then filtered with a 0.2 μm Millipore filter and adsorption spectra were obtained for each extract using a HPA-8452A UV-Visible Diode Array Spectrophotometer from 320 to 750 nm. Absorbance values at 470 nm (max. for carotenoids), 647 nm (max. for chlorophyll *b*), and 663 nm (max. for chlorophyll *a*) were selected for semiquantitative determination of pigment contents using the trichromatic equations and extinction coefficients previously reported by (Lichtenthaler, 1987). The absorbance value at 750 nm was subtracted from all measured absorbances. The final values were the averages of four independent determinations (± standard deviation) (Table S2).

**Table S2.** Selected pigment contents within the cryptoendolithic orange and green zone

|  |  | Pigments [mg / gW] | |  |  |
| --- | --- | --- | --- | --- | --- |
| *Sampling zone* | **Chl *a*** | **Chl *b*** | **Chl *a* + *b*** | **Carotenoids** | **Chl *a*+*b* / Car** |
| Orange | 10.949 | 4.713 | 15.662 | 12.073 | 1.297 |
| Green | 12.737 | 8.032 | 20.769 | 5.181 | 4.009 |

**S8. Analytical results**

**S8.1. Microclimate parameters evolution**


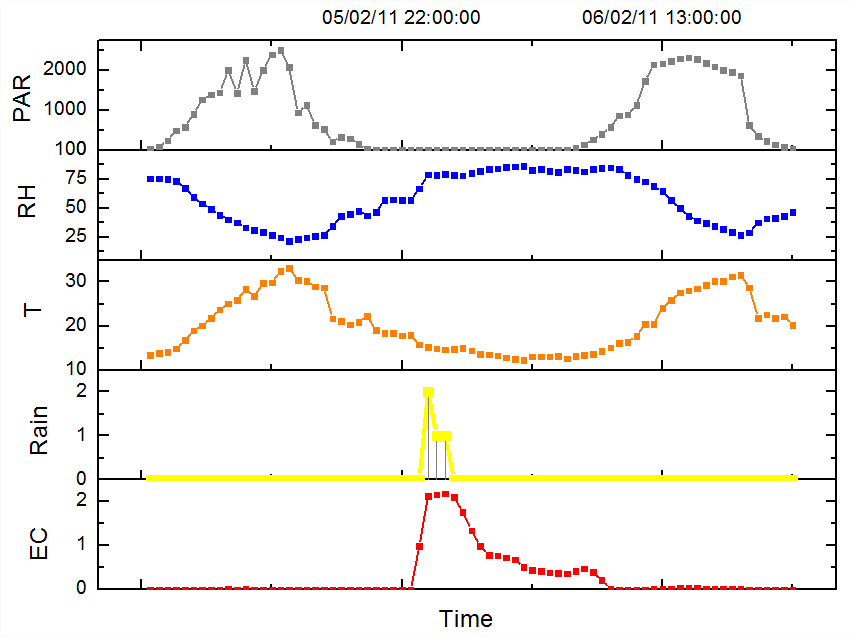


**Figure S1.** Microclimatic parameters (PAR [μmol photons m^2^ s^-1^], RH [%], T [ºC], rain [mm] and EC [V]) over 37 hours on February 5/6, 2011 with a recorded rainfall event. The graph starts on February 5, 2011 at 7:30 a.m. Changes in PAR indicated the presence of scattered clouds during the day-time with a maximum PAR of 2506 μmol photons m^2^ s^-1^.

**S8.2. Sepiolite HRTEM-EDS analysis**


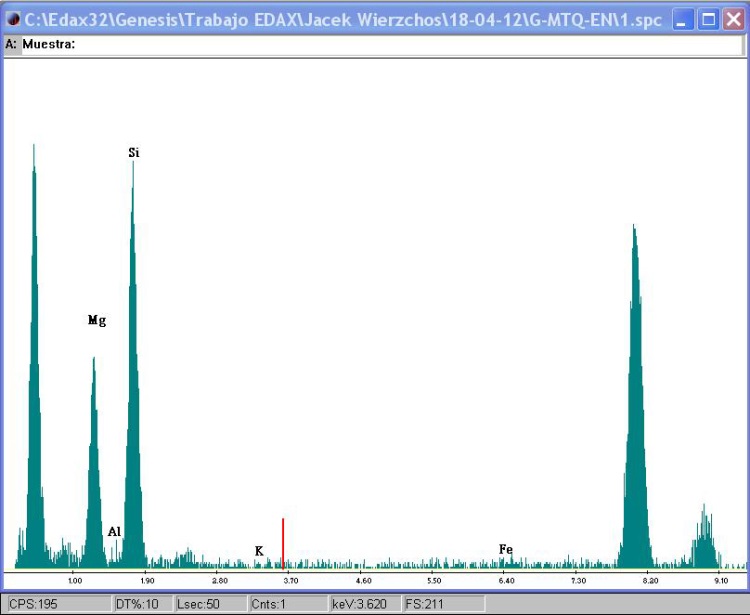
**Figure S2.** Energy dispersive X-ray spectrum of sepiolite from clay minerals attached to cyanobacteria aggregates and isolated below the algal cryptoendolithic layer. Data was visualized by HRTEM. A sepiolite analytical standard was used to perform quantitative identification of sepiolite minerals.

**S8.3. Molecular characterization of endoliths**

Colonized zone from rock substrates were harvested with sterilized drill bits and a Dremel power tool (DiRuggiero et al., 2013). Total genomic DNA from rock powder was extracted using the PowerSoil DNA Isolation kit (MoBio laboratories Inc., Solana Beach, CA) according to protocols previously optimized (Robinson et al., 2015; DiRuggiero et al., 2013; Crits-Christoph et al., 2013). Extractions were carried out in triplicate on all samples and the resulting nucleic acids pooled for better representation. All sample manipulations and extractions were performed in a laminar flow hood (AirClean Systems, Creedmoor, NC) and all materials and reagents were either filter sterilized, autoclaved, or UV irradiated to prevent contamination. DNA was amplified using the barcoded universal primers 27F and 506R for the V1–V3 hypervariable region of the 16S rRNA gene and amplicons from 3 reactions were pooled together for sequencing, using the Illumina MiSeq plateform, by the Genomics Resource Center (GRC) at the Institute for Genome Sciences (IGS), University of Maryland School of Medicine (Robinson et al., 2015). For data analysis, we used the same strategy as for our previous analyses of soil and endoliths from the Atacama Desert (Crits-Christoph et al., 2013; DiRuggiero et al., 2013; Robinson et al., 2015). For this, Illumina paired-end sequences were assembled using PANDAseq (Masella et al., 2012) and further processed using the QIIME package (v1.6.0) (Caporaso et al., 2010). Sequences were clustered together using USEARCH (Edgar et al., 2010) and de novo chimera detection conducted in UCHIME v5.1 (Edgar et al., 2010). Taxonomic ranks were assigned using the appropriate classifier and databases. Richness and diversity estimators were calculated based on OTUs with QIIME (Caporaso et al. 2010). Statistical analyses were performed using the R statistical package (<http://www.R-project.org>.), non-parametric Kruskal–Wallis one-way analyses of variance (Kruskal et al., 1952), and Least Squares Linear Regression. Phylogenetic trees were built using MEGA 6.06 (Tamura et al., 2011;Robinson et al., 2015).

**Table S3.** Observed richness and diversity indices for the gypsum community from two rock samples based on 16S rRNA genes sequences and assignments with a 97% sequence similarity threshold. Dataset were of equal size and subsampled at 1,662 sequence reads; average of 10 iterations.

|  | OTUs observed richness | Shannon diversity index | Faith’s phylogenetic diversity index | Chao1 diversity estimate | Pielou’s evenness index |
| --- | --- | --- | --- | --- | --- |
| CL1 | 260± 11 | 5.4 ± 0.07 | 14.8 ± 0.7 | 588 ± 58 | 0.68 ± 0.007 |
| CL2 | 167 ± 7 | 5.7 ± 0.06 | 11.9 ± 0.5 | 268 ± 45 | 0.77 ± 0.006 |

**Figure S3.** Evolutionary relationship of major OTUs from the gypsum community inferred using the maximum likelihood method as implemented by MEGA 6.06 using 16s rRNA sequences (428 positions). All positions containing gaps and missing data were eliminated. Strain names are followed by geographic location of collection and preceded by GenBank accession numbers. Bar represent 0.02% sequence divergence. Bootstrap values above 50% (1000 replicates) are shown at nodes. Open diamonds, OTUs present only in sample CL1; open circles, OTUs present only in sample CL2; close diamonds, OTUs present in both samples.

**S8.4. Raman spectroscopy analyses**

Point measurements were replicated on several spots within the studied colonization zones. The Raman microspectrometer was equipped with a microscope with a standard 50x objective and a long-working distance objective (50x). The laser power was typically set to 3–15 mW using 785 nm excitation and 1–2 mW using 532 nm laser excitation to obtain an optimal Raman signal and simultaneously avoid any thermal alteration of the sample. Typically, 15 s scans were accumulated 1–20 times at 785 nm and 0.5-2 s scans accumulated 20-40 times at 532 nm. The parameters were dependent on the signal-to-noise ratio from the particular areas.

For Raman imaging of carotenoids within the algal zone, a rock transects flattened by knife was analysed. A Renishaw InVia spectrometer with a streamline mode (linefocus) was employed. 5x magnification objective and Ar laser line at 514.5 nm were used with 2.5 mW power at source and 12 s exposure time. Imaging data were processed using the Wire 3.4 software allowing chemometric data processing. First, spectral artefacts due to cosmic rays were removed using the nearest neighbour method and the spectral noise was filtered through the datasets before map construction. The image map was created as “signal-to-baseline” intensity in the region of 1500-1535 cm^-1^, corresponding to the occurrence of ν_1_(C=C) band. LUT (Look Up Table) was set to obtain optimal contrast. Individual Raman spectra from different areas of the map were checked and examples extracted from the imaging datasets are shown in Figure S5.


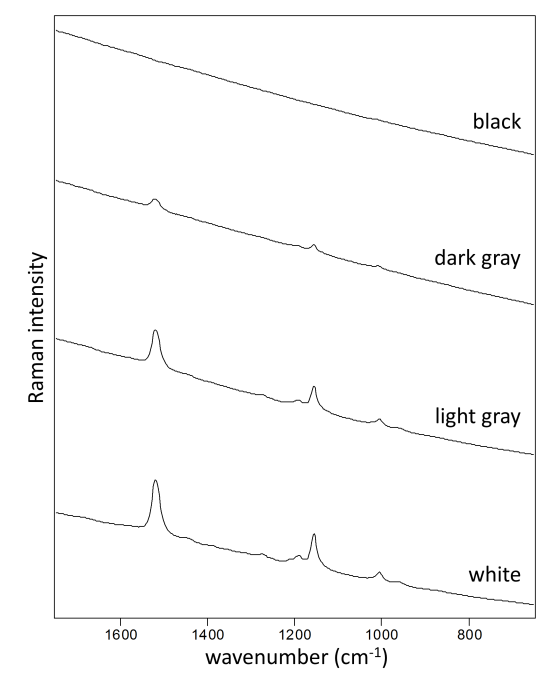


**Figure S5.** Individual spectra extracted from the different areas of a Raman distribution map (in text Fig. 11f) constructed based on signal-to-baseline of ν_1_(C=C) carotenoid band in the region of 1500 – 1535 cm^-1^.

**S8.4.1. Raman identification of carotenoids**

The differences between green and orange algal cells and cyanobacterial Raman signals are depicted in Fig. S6. The carotenoids were identified by the strong bands corresponding to ν_1_(C=C) and ν_2_(C-C) stretching vibrations and a feature of medium intensity that occurs around 1000 - 1008 cm^−1^, corresponding to the in-plane rocking modes of the CH_3_ groups attached to the polyene chain (Merlin, 1985). The ν_1_ and ν_2_ bands here are located at 1513 – 1524 cm^-1^ (Fig. S6) and 1155 – 1157 cm^-1^, respectively. Especially, the position of the ν_1_(C=C) band is indicative of different carotenoid structures based on the conjugated skeletal chain length, molecular termination or differences of bonding to the cell structures and is also slightly dependent on the excitation wavelength of the laser used for analysis (Ruban et al., 2001; Andreeva and Velitchkova 2005; de Oliveira et al., 2010). Moreover, as more than one carotenoids are usually present within the cell of phototrophic microorganisms, selective enhancement of some carotenoids may occur when the excitation wavelength coincides with the absorption band of an allowed π−π* electronic transition (Vítek et al., 2013). This coincidence results in the resonance Raman effect, which significantly enhances the intensity of the Raman signal (Gill et al., 1970, Merlin, 1985, Withnall et al., 2003; Marshall et al., 2007). Due to this resonance Raman effect, generally the most favourable excitation wavelength for the identification of carotenoids lies around 500 nm (e.g. 488 nm, 514.5 nm, 532 nm) (Withnall et al., 2003; Marshall et al., 2007). Raman signal intensity of particular carotenoid with the best coincidence with the used laser wavelength may be selectively enhanced relatively to other carotenoids (see comparison of 785 nm and 514.5 nm excitation used for analysis of cyanobacteria in Vítek et al., 2013).

**
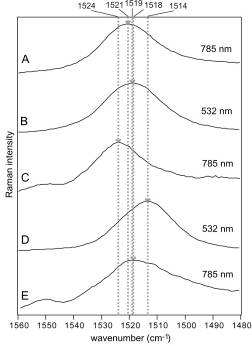
**

**Figure S6**. Differences in carotenoid ν_1_(C=C) positions as obtained on red/orange algal cells (A, B) and green algal cells (C, D) using 785 nm (A, C) and 532 nm laser line for excitation, respectively (B, D). The spectra (E) represents the carotenoid ν_1_(C=C) position of cyanobacterial carotene.

**S9. References**

Andreeva, A., and Velitchkova, M. (2005). Resonance Raman spectroscopy of carotenoids in Photosystem I particles. *Bioph. Chem.* 114, 129-135.

Boison, G., Mergel, A., Jolkver, H., and Bothe, H. (2004). Bacterial life and dinitrogen fixation at a gypsum rock. *Appl. Environ. Microbiol.* 70, 7070-7077. doi: 10.1128/AEM.70.12.7070-7077.2004

Braslavsky, S.E. (2007). Glossary of terms used in photochemistry, 3^rd^ edition (IUPAC Recommendations 2006). *Pure and App. Chem.* 79, 293-465.

Cámara, B. (2012). Colonización microbiana de yeso e ignimbrita en la región hiperárida del Desierto de Atacama. Universidad Autónoma de Madrid, 1-244.

Caporaso, J. G., C. L. Lauber, W. A. Walters, D. Berg-Lyons, C. A. Lozupone, P. J. Turnbaugh, N. Fierer, and Knight R. (2011). Global patterns of 16S rRNA diversity at a depth of millions of sequences per sample. Proc Natl Acad Sci USA 108:4516-4522.

Chung, F.H. (1974). Quantitative interpretation of X-ray-diffraction patterns of mixtures. Adiabatic principle of x-ray-diffraction analysis of mixtures. *J. App. Crystall.* 7, 526-531.

Cockell, C.S., Osinski, G.R., Banerjee, N.R., Howard, K.T., Gilmour, I., and Watson, J.S. (2010). The microbe-mineral environment and gypsum neogenesis in a weathered polar evaporite. *Geobiology* 8: 293-308. doi: 10.1111/j.1472-4669.2010.00240.x.

Crits-Christoph, A., C. K. Robinson, T. Barnum, W. F. Fricke, A. F. Davila, B. Jedynak, C. McKay, and DiRuggiero, J.. (2013). Colonization patterns of soil microbial communities in the Atacama Desert. Microbiome 1:28.

de Oliveira, V. E., Castro, H. S., Edwards, H. G. M., de Oliveira, L. F. C. (2010) Carotenes and carotenoids in natural biological samples: a Raman spectroscopic analysis. *Journal of Raman Spectroscopy* 41: 642-650.

DiRuggiero, J., Wierzchos, J., Robinson, C.K., Souterre, T., Ravel, J., Artieda, O., Souza-Egipsy, V., and Ascaso, C. (2013). Microbial colonisation of chasmoendolithic habitats in the hyper-arid zone of the Atacama Desert. *Biogeosciences* 10: 2439-2450. doi: 10.5194/bg-10-2439-2013.

Dong, H., Rech, J.A., Jiang, H., Sun, H., and Buck, B.J. (2007). Endolithnic cyanobacteria in soil gypsum: Occurences in Atacama (Chile), Mojave (United States), and Al-Jafr Basin (Jordan) Deserts. *Journal of Geophysical Research G: Biogeosciences* 112.

Edgar, R. C. (2010). Search and clustering orders of magnitude faster than BLAST. Bioinformatics 26:2460-2461.

Edwards, H.G.M., Villar, S.E.J., Parnell, J., Cockell, C.S., and Lee, P. (2005). Raman spectroscopic analysis of cyanobacterial gypsum halotrophs and relevance for sulfate deposits on Mars. *Analyst* 130: 917-923. doi: 10.1039/b503533c.

Gill, D., Kilponen, R. G., and Rimai, L. (1970) Resonance Raman scattering of laser radiation by vibrational modes of carotenoid pigment molecules in intact plant tissues. *Nature* 227: 743.

Lichtenthaler, K. 1987. Pigments of photosynthetic biomembranes. *Methods Enzymol.* 148: 366–94.

Hughes, K.A., and Lawley, B. (2003). A novel Antarctic microbial endolithic community within gypsum crusts. *Environmental microbiology* 5: 555-565.

Kruskal, W. H., and Wallis, W. A. (1952). Use of ranks in one-criterion variance analysis. J Am Stat Assoc 46:583–621.

Marshall, C. P., Leuko, S., Coyle, C. M., Walter, M. R., Burns, B. P., and Neilan, B. A. (2007) Carotenoid Analysis of Halophilic Archaea by Resonance Raman Spectroscopy. *Astrobiology* 7: 631-643.

Masella, A. P., A. K. Bartram, J. M. Truszkowski, D. G. Brown, and Neufeld, J. D. (2012). PANDAseq: paired-end assembler for illumina sequences. BMC Bioinformatics 13:31.

Merlin, J. C. (1985) Resonance Raman spectroscopy of carotenoids and carotenoid-containing systems. *Pure and Applied Chemistry* 57: 785–792.

Omelon, C.R., Pollard, W.H., and Ferris, F.G. (2006) Environmental controls on microbial colonization of high Arctic cryptoendolithic habitats. *Polar Biology* 30: 19-29.

Parnell, J., Lee, P., Cockell, C.S., and Osinski, G.R. (2004). Microbial colonization in impact-generated hydrothermal sulphate deposits, Haughton impact structure, and implications for sulphates on Mars. *International Journal of Astrobiology* 3: 247-256. doi: doi:10.1017/S1473550404001995.

Robinson, C.K., Wierzchos, J., Black, C., Crits-Christoph, A., Ma, B., Ravel, J. et al. (2015) Microbial diversity and the presence of algae in halite endolithic communities are correlated to atmospheric moisture in the hyper-arid zone of the Atacama Desert. *Environ Microbiol*. 17: 299-315.

Rhind, T., Ronholm, J., Berg, B., Mann, P., Applin, D., Stromberg, J., Sharma, R., Whyte, L.G., and Cloutis, E.A. (2014). Gypsum-hosted endolithic communities of the Lake St. Martin impact structure, Manitoba, Canada: spectroscopic detectability and implications for Mars. *International Journal of Astrobiology* 13: 366-377. doi: doi:10.1017/S1473550414000378.

Ruban, A. V., Pascal, A. A., Robert, B., and Horton, P. (2001) Configuration and dynamics of xantophylls in light harvesting antennae of higher plants. *Journal of Biological Chemistry* 276: 24862-24870.

Stivaletta, N., and Barbieri, R. (2009). Endolithic microorganisms from spring mound evaporite deposits (southern Tunisia). *Journal of Arid Environments* 73: 33-39. doi: 10.1016/j.jaridenv.2008.09.024.

Stivaletta, N., López-García, P., Boihem, L., Millie, D.F., and Barbieri, R. (2010). Biomarkers of Endolithic Communities within Gypsum Crusts (Southern Tunisia). *Geomicrobiology Journal* 27: 101-110. doi: 10.1080/01490450903410431.

Tamura, K., D. Peterson, N. Peterson, G. Stecher, M. Nei, and Kumar, S. (2011). MEGA5: Molecular Evolutionary Genetics Analysis using Maximum Likelihood, Evolutionary Distance, and Maximum Parsimony Methods. Mol Biol Evol 28:2731-2739.

Vítek, P., Cámara-Gallego, B., Edwards, H.G.M., Jehlička, J., Ascaso, C., and Wierzchos, J. (2013). Phototrophic Community in Gypsum Crust from the Atacama Desert Studied by Raman Spectroscopy and Microscopic Imaging. *Geomicrobiology Journal* 30: 399-410.

Wierzchos, J., Cámara, B., De Los Ríos, A., Davila, A.F., Sánchez Almazo, I.M., Artieda, O., Wierzchos, K., Gómez-Silva, B., Mckay, C., and Ascaso, C. (2011). Microbial colonization of Ca-sulfate crusts in the hyperarid core of the Atacama Desert: Implications for the search for life on Mars. *Geobiology* 9: 44-60.

Wierzchos, J., Davila, A.F., Sánchez-Almazo, I.M., Hajnos, M., Swieboda, R., and Ascaso, C. (2012) Novel water source for endolithic life in the hyperarid core of the Atacama Desert. *Biogeosciences* 9: 2275-2286.

Wierzchos, J., Davila, A.F., Artieda, O., Cámara-Gallego, B., de los Ríos, A., Nealson, K.H. et al. (2013) Ignimbrite as a substrate for endolithic life in the hyper-arid Atacama Desert: Implications for the search for life on Mars. *Icarus* 224: 334-346.

Withnall, R., Chowdhry, B. Z., Silver, J., Edwards, H. G. M., and de Oliveira, L. F. C. (2003) Raman spectra of carotenoids in natural products. *Spectrochimica Acta A* 59: 2207–2212.

Ziolkowski, L.A., Mykytczuk, N.C.S., Omelon, C.R., Johnson, H., Whyte, L.G., and Slater, G.F. (2013a). Arctic gypsum endoliths: A biogeochemical characterization of a viable and active microbial community. *Biogeosciences* 10: 7661-7675. doi: 10.5194/bg-10-7661-2013.

Ziolkowski, L.A., Wierzchos, J., Davila, A.F., and Slater, G.F. (2013b). Radiocarbon evidence of active endolithic microbial communities in the hyperarid core of the atacama desert. *Astrobiology* 13: 607-616. doi: 10.1089/ast.2012.0854.
